# Latent signal models: Learning compact representations of signal evolution for improved time-resolved, multi-contrast MRI

**DOI:** 10.1002/mrm.29657

**Published:** 2023-04-24

**Authors:** Yamin Arefeen, Junshen Xu, Molin Zhang, Zijing Dong, Fuyixue Wang, Jacob White, Berkin Bilgic, Elfar Adalsteinsson

**Affiliations:** 1 Department of Electrical Engineering and Computer Science, Massachusetts Institute of Technology, Cambridge, Massachusetts, USA; 2 Athinoula A. Martinos Center for Biomedical Imaging, Charlestown, Massachusetts, USA; 3 Department of Radiology, Harvard Medical School, Boston, Massachusetts, USA; 4 Harvard-MIT Health Sciences and Technology, Massachusetts Institute of Technology, Cambridge, Massachusetts, USA; 5 Institute for Medical Engineering and Science, Massachusetts Institute of Technology, Cambridge, Massachusetts, USA

**Keywords:** image reconstruction, machine learning, model-based, time-resolved MRI

## Abstract

**Purpose:**

To improve time-resolved reconstructions by training auto-encoders to learn compact representations of Bloch-simulated signal evolution and inserting the decoder into the forward model.

**Methods:**

Building on model-based nonlinear and linear subspace techniques, we train auto-encoders on dictionaries of simulated signal evolution to learn compact, nonlinear, latent representations. The proposed latent signal model framework inserts the decoder portion of the auto-encoder into the forward model and directly reconstructs the latent representation. Latent signal models essentially serve as a proxy for fast and feasible differentiation through the Bloch equations used to simulate signal. This work performs experiments in the context of T_2_-shuffling, gradient echo EPTI, and MPRAGE-shuffling. We compare how efficiently auto-encoders represent signal evolution in comparison to linear subspaces. Simulation and in vivo experiments then evaluate if reducing degrees of freedom by incorporating our proxy for the Bloch equations, the decoder portion of the auto-encoder, into the forward model improves reconstructions in comparison to subspace constraints.

**Results:**

An auto-encoder with 1 real latent variable represents single-tissue fast spin echo, EPTI, and MPRAGE signal evolution to within 0.15% normalized RMS error, enabling reconstruction problems with 3 degrees of freedom per voxel (real latent variable + complex scaling) in comparison to linear models with 4–8 degrees of freedom per voxel. In simulated/in vivo T_2_-shuffling and in vivo EPTI experiments, the proposed framework achieves consistent quantitative normalized RMS error improvement over linear approaches. From qualitative evaluation, the proposed approach yields images with reduced blurring and noise amplification in MPRAGE-shuffling experiments.

**Conclusion:**

Directly solving for nonlinear latent representations of signal evolution improves time-resolved MRI reconstructions.

## 1 | INTRODUCTION

Efficient time-resolved MRI enables a wide range of applications such as MRS,^1^ quantitative parameter mapping,^2,3^ motion-resolved imaging,^4,5^ and blur-free, multi-contrast imaging from fast-spin-echo^6^ (FSE) and MPRAGE^7,8^ acquisitions. In this paper, we focus on applications that reconstruct individual echo images with differing contrasts from acquisitions with evolving signal evolution over an echo train.^7^

Time-resolved MRI requires lengthy scan times using traditional methods. Compressed sensing^9,10^ and parallel imaging^11,12^ reduce acquisition times in structural MRI, with some time-resolved applications.^13^ Machine learning provides another avenue for acceleration. Models, typically neural networks trained in a supervised fashion, impose custom regularization or directly reconstruct undersampled data.^14–18^ Some work demonstrates applications of supervised and unsupervised machine learning in dynamic imaging,^19,20^ but the cost of acquiring fully sampled data hampers widespread use of supervised learning in many time-resolved acquisitions.

Time-resolved MRI employs a variety of different sequence and reconstruction techniques to reduce acquisition times in the presence of the additional imaging dimension. Some techniques model signals with an analytic formula and then resolve the underlying tissue parameters that characterize this model,^21–26^ introducing a nonlinear optimization problem that may not account for slice-profile effects, stimulated echoes, and B1+ inhomogeneity.^27^ To model these effects, more complicated techniques build the Bloch equations into the forward model and solve for the underlying parameters governing the model^28–30^ but require extensive computation or approximation of gradients.

Alternatively, linear subspace–based constraints exploit temporal redundancies to significantly reduce the degrees of freedom in the reconstruction problem.^3,6,31–35^ Low-rank constraints^3,36,37^ combined with compressed sensing^9,10^ and parallel imaging,^11,12^ also synergistically improve reconstruction quality.

For example, the highly undersampled shuffling^6,7,38,39^ and echo-planar-time-resolved-imaging^2,40^ (EPTI) techniques resolve multi-contrast images from FSE^40^, MPRAGE, and EPI acquisitions. These techniques simulate dictionaries of signal evolution and generate linear subspaces using the singular value decomposition (SVD). The forward models explicitly incorporate subspace constraints and directly solve for (and impose regularization^36^ on) the associated unknown linear coefficients with significantly reduced degrees of freedom. Shuffling reconstructs multi-contrast images with improved sharpness as signal evolutions no longer modulate k-space. Similarly, EPTI produces distortion- and blurring-free images across the EPI readout. Shuffling yields positive clinical outcomes,^39^ whereas EPTI enables distortion-/blurring-free, rapid quantitative mapping, diffusion imaging,^42^ and fMRI.^43^

However, linearization of the nonlinear reconstruction problem may induce more degrees of freedom than what exists in the underlying physical process. Additionally, linearization requires solving for both the real and imaginary portion of each complex linear coefficient, further increasing the number of unknowns.

Recently, auto-encoders trained on dictionaries of simulated signal in MRI spectroscopy and diffusion acquisitions have learned compact latent representations of signal space.^44,45^ The proposed MRSI and diffusion techniques employ the trained auto-encoders as regularization in reconstruction.

We propose latent signal models, combining ideas from Bloch-equation models, linear subspaces, and latent representations for improved time-resolved MRI reconstruction. Latent signal models simulate dictionaries of signal evolution to train an auto-encoder to learn a compact representation of signal. The proposed reconstruction framework then incorporates the decoder portion of the trained auto-encoder into the imaging forward model and solves for the learned latent representation of signal. This improves reconstruction quality with reduced degrees of freedom in comparison to linear techniques in the presence of undersampling. The decoder produces the time-series of multi-contrast images from the reconstructed latent representation. Latent signal models learn to represent the Bloch equations with a simple neural network that can be inserted into the forward model-enabling reconstruction with the sophisticated auto-differentiation tools developed for machine learning.^46^ In this way, we feasibly, quickly, and conveniently solve a reconstruction problem that maintains the benefits of and also circumvents the demanding optimization usually induced by incorporating the Bloch equations into the forward model.

We begin by introducing and characterizing our latent signal model framework through application in T_2_-shuffling. We show that auto-encoders learn more compact representations of single-tissue FSE signal evolution in comparison to linear models. Then, simulation and retrospectively and prospectively undersampled in vivo reconstruction experiments suggest that the reduced degrees of freedom afforded by the latent signal model framework improves reconstruction quality in comparison to linear subspace constraints. Additional experiments analyze the stability of the technique across varying noise instances. Next, we empirically verify that the trained auto-encoder essentially serves as a fast and feasible proxy for optimization through the simulation of signal evolution. We further demonstrate versatility through improved reconstructions in in vivo, retrospectively undersampled gradient echo (GE)-EPTI, and undersampled MPRAGE-shuffling experiments. Finally, we characterize our proposed approach in the presence of multi-tissue partial volumes.

## 2 | METHODS

### 2.1 | Time-resolved MRI with linear subspace constraints

Sequences that acquire multiple k-space lines after excitation or inversion across a GE or spin-echo train concatenate data from all echoes into a single time-point, often of dimension M×N×P×C, where M,N,P represent read-out, phase-encode, and partition dimensions, respectively; and C represents the number of coils. Reconstructions produce a static image that suffers from blurring^47^ or distortion^48^ artifacts due to signal decay and phase evolution in the collapsed temporal evolution.

To improve image sharpness and reduce distortion, techniques like EPTI and shuffling aim to resolve $x\in C^{M\times N\times P\times T}$, a time-series of multi-contrast images, where T represents the number of echoes or timepoints, by considering the temporal representation of acquired k-space, $y\in C^{M\times N\times P\times C\times T}$, which assigns k-space points to their associated time of acquisition.^2,6^ However, the resultant reconstruction problems become heavily underdetermined because resolving the time dimension increases the number of unknowns by a factor of T.

To produce tractable reconstruction problems, linear subspace techniques generate a dictionary, D, of simulated signal evolution from realistic tissue relaxation parameters for the desired sequence of interest. Then the first B singular vectors of D form a low dimensional subspace $\Phi \in C^{T\times B}$ that can be inserted into the forward model to produce:

(1) $$\alpha_{*}=\underset{\alpha }{argmin}\|y-F\;S\;H\;\Phi \;\alpha \|+\lambda \;R(\alpha ),$$

where F and S represent the undersampled Fourier and Coil–sensitivity operators applied to each time-point; H represents temporally varying phase (if necessary); $\alpha \in C^{B\times (MNP)}$ represents the subspace coefficients the optimization problem solves for; and R represents a spatial-regularization function applied to the subspace coefficients. The estimated coefficients $\alpha_{*}$ yields the time-series of images with $x_{*}=\Phi \alpha_{*}$. This formulation reduces the number of unknowns by $\frac{T}{B}$. For example, some applications of T_2_-shuffling reconstruct data from an FSE sequence with T=80 and utilize subspaces with B=4, reducing unknowns by a factor of 20×.

### 2.2 | Latent signal models reconstruction framework for time-resolved MRI

We propose learning a nonlinear latent representation of the signal evolution dictionary, D. Let $E_{\Theta }$ and $Q_{\Psi }$ represent fully connected neural networks for the encoder and decoder of an auto-encoder.^49,50^ The auto-encoder learns a latent representation of signal evolution by minimizing the following with respect to its weights and biases, Θ and Ψ:

(2) $$\Theta^{*},\Psi^{*}=\underset{\Theta ,\Psi }{argmin}{\left\|D-Q_{\Psi }\left(E_{\Theta }(D)\right)\right\|}_{2}^{2}.$$

**FIGURE 1:**
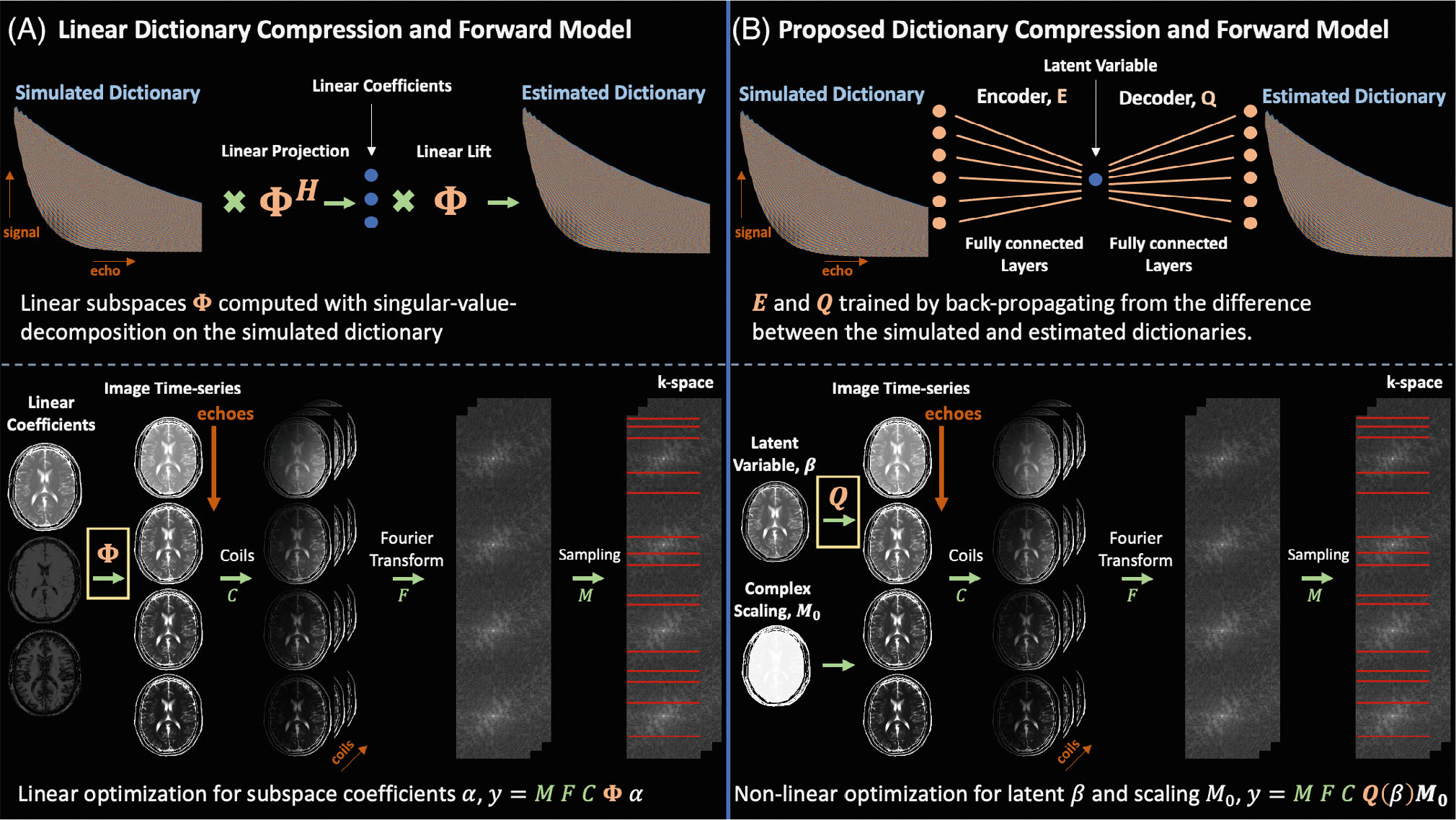
Comparing linear subspace constraints and the proposed latent signal model framework in the T_2_-shuffling setting. To represent FSE signal evolution, (A) the SVD generates a subspace from a dictionary of signal evolution with 2–4 complex coefficients (resulting in {4,6,8} degrees of freedom per voxel in the reconstruction problem), whereas (B) the auto-encoder learns a more compact representation of signal evolution with 1 real latent variable (resulting in 3 degrees of freedom per voxel in the reconstruction problem). Inserting either the subspace or decoder in the forward model reduces the number of variables required to resolve signal dynamics. The auto-encoder’s more compact latent representation, compared to linear subspaces, enables improved reconstruction quality in subsequent experiments. FSE, fast spin echo; SVD, singular value decomposition.

The trained decoder can then be inserted in the forward model,

(3) $$\beta^{*},M_{0}^{*}=\underset{M_{0},\rho }{argmin}{\left\|y-F\;S\;H\left[M_{0}Q_{\Psi^{*}}(\beta )\right]\right\|}_{2}^{2}\;\;+\lambda \;R\left(\beta ,M_{0}\right)$$

to reconstruct the latent representation $\beta^{*}\in R^{M\times N\times P\times L}$, complex scaling $M_{0}^{*}\in C^{M\times N\times P}$, and time-series of images with $x^{*}=M_{0}^{*}Q_{\Psi^{*}}(\beta^{*})$, where L is the number of latent variables. Unlike previous work,^44,45^ the decoder prior does not require tuning an additional regularization parameter, and separate spatial regularization can be applied directly to β and $M_{0}$. In essence, the decoder neural network serves as a proxy for the simulation process used to generate the dictionary of signal evolution and synergizes with auto-differentiation tools for fast and feasible optimization. Figure 1 visualizes the latent signal model framework in comparison to the standard linear approach through an exemplar T_2_-shuffling setting.

### 2.3 | Comparing degrees of freedom in latent signal models and linear reconstructions

With complex-valued coefficients α, linear reconstructions resolve M×N×P×B×2 unknowns, whereas the real-valued latent representation β and the complex-valued scaling $M_{0}$ yield M×N×P×(L+2) unknowns in the proposed framework. If L+2<2×B the latent signal model framework produces a reconstruction problem with fewer unknowns.

### 2.4 | T_2_-shuffling experiments

Experiments begin in T_2_-shuffling^6^ settings, whereas subsequent results showcase the technique’s versatility through GE-EPTI and MPRAGE-shuffling.

#### 2.4.1 | Dictionary compression

We compare how efficiently SVD-generated linear subspaces and a trained auto-encoder represent single-tissue FSE signal evolution. Extend-phase-graph (EPG) simulations^51^ generated training and testing dictionaries of signal evolution for T_2_ = 50–400 ms, T_1_ = 1000 ms, 80 echoes (*T* = 80), 160-degree refocusing pulses, and 5.56 ms echo spacing. From the training dataset, SVD generated linear subspaces with {1,2,3,4} singular vectors (resulting in reconstruction problems with {2,4,6,8} degrees of freedom per voxel), whereas minimizing Equation (3) produced auto-encoders with {1,2,3,4} latent variables (resulting in reconstruction problems with {3,4,5,6} degrees of freedom per voxel).

Each auto-encoder utilized two fully connected layers for both the decoder and encoder with hyperbolic tangent^52^ (tanh) activation functions. T×1 vectors representing signal evolutions served as inputs to the auto-encoder. The first layer of the encoder transforms the T×1 input vector to $\frac{T}{2}\times 1$, and the second layer takes the $\frac{T}{2}\times 1$ vector to the latent L×1 representation. The decoder performs the same operations, characterized by different weights, in reverse. Linear subspaces and auto-encoders compressed and reconstructed signal in the testing dictionary, and the resultant reconstruction accuracies were compared.

#### 2.4.2 | Simulated reconstruction experiments

Next, we simulated T_2_-shuffling acquisitions from a numerical phantom with single-tissue T_1_, T_2_, $M_{0}$, and 8-channel coil-sensitivity maps.^53^ EPG simulations produced a time-series of k-spaces (with added noise) for each echo of a FSE sequence: T=80 echoes, 5.56 ms echo spacing, and 160-degree refocusing pulses. We applied an undersampling mask that mimics a 2D T_2_-shuffling acquisition with four shots that samples a random phase-encode line at each echo and labeled the dataset *simulated_t2shfl1*. This corresponds to 320 sampled k-space lines, resulting in an overall acceleration factor with respect to the desired time-series of images to reconstruct of $R=\frac{256*80}{320}=64$.

The experiment compares linear subspace reconstructions using {4,6} degrees of freedom (or {2,3} complex coefficients) and wavelet or locally low-rank regularization to the proposed latent signal model reconstruction with 3 degrees of freedom and wavelet regularization (1 latent variable, 1 real $M_{0}$, 1 imaginary $M_{0}$). The regularization parameter, λ, in all reconstructions was experimentally optimized to minimize error. Auto-differentiation in PyTorch^54^ with a GPU-compatible implementation of Wavelets^55^ solved the proposed reconstruction, whereas the Berkeley Advanced Reconstruction Toolbox (BART)^56^ toolbox reconstructed the linear problem.

#### 2.4.3 | Retrospective in vivo reconstruction experiments

All imaging protocols were performed with approval from the local institutional review board with written informed consent. A volunteer was scanned with a Siemens 3 T Trio (Siemens Healthineers, Erlangen, Germany) system using a 12-channel receive head coil. A fully sampled spatial and temporal multi-echo T_2_-weighted dataset with FOV = 180 mm × 240 mm, matrix size = 208 × 256 matrix size, slice thickness = 3 mm, echo spacing = 11.5 ms, and T=32 echoes was acquired.

The applied undersampling mask generated acquisitions with seven shots that sample a random phase-encode line at each echo, and we label the dataset *invivo_t2shfl1*. This corresponds to 224 sampled k-space lines, resulting in an overall acceleration of $R=\frac{256*32}{224}\approx 36$.

The experiment compares linear subspace reconstructions with {4,6} degrees of freedom and wavelet or locally low-rank regularization to the proposed latent signal model reconstruction with 3 degrees of freedom and wavelet regularization. Additionally, the proposed approach utilized an auto-encoder with three fully connected layers and tanh activation functions. We chose all experimentally optimized regularization parameters to minimize error, and the auto-differentiation and BART frameworks solved the proposed and linear problems, respectively.

#### 2.4.4 | Reconstruction stability across various noise instances

The aforementioned experiments combined temporal constraints, spatial regularization, and tuned hyperparameters for best reconstruction performance. Thus, the following experiment isolates and characterizes the improvements afforded by the reduced degrees of freedom from latent signal models and evaluates stability of the reconstruction across various noise instances in the spirit of g-factor^12^ analysis.

With identical sequence parameters, we generated an additional dataset, labeled *simulated_t2shfl2*, modeling a 3D T_2_-shuffling acquisition (with single-tissue voxels) by assuming each echo–k-space corresponds to phase- and partition-encode dimensions and sampling 256 × 256 k-space points randomly throughout the 80 echoes.^6^ We added 250 instances of realistic Gaussian noise to the *simulated_t2shfl2* dataset and 2D-, seven-shot, in vivo *invivo_t2shfl1* dataset. Then, the proposed latent signal model framework with 3 and linear subspaces with {4,6} degrees of freedom reconstructed the 250 k-spaces in both the simulation and retrospective regimes without regularization. We report the mean and SD of the normalized RMS error (NRMSE) at each reconstructed TE time and display mean absolute error maps.

#### 2.4.5 | Learning a proxy for differentiating through MRI simulations in a reconstruction setting

The subsequent experiment demonstrates that the trained auto-encoder serves as a proxy for fast and feasible optimization through the signal simulation process directly in the reconstruction problem.

Let G represent the simulation function that produces signal evolution given an input T_2_ and $M_{0}$ in T_2-_shuffling. The simulation-based reconstruction problem becomes:

(4) $$\underset{T_{2},\rho }{argmin}V\left(T_{2},\rho \right)={\left\|y-F\;S\;H\;G\left(T_{2},M_{0}\right)\right\|}_{2}^{2},$$

which solves for the $T_{2}\in R^{M\times N}$ map and scaling $M_{0}\in C^{M\times N}$.

We show that minimizing Equation (3) in the latent signal model framework produces a solution that also minimizes the simulation-based reconstruction problem in Equation (4). We use the proposed framework to reconstruct the *simulated_t2shfl2* datasets and 3D retrospectively undersampled in vivo dataset with R = 32 (both without regularization). The optimization runs for 1000 iterations, applying the following procedure every 50 iterations:

Extract the current latent variable and scaling estimates, $\beta_{i}$ and $M_{0,i}$.

• With $\beta_{i}$, estimate a T_2_ map, $T_{2,i}$, using dictionary matching directly in latent variable space.^57^

Compute and record the $L_{2}$ norms of the following gradients ${\left\|\nabla_{T_{2}}\left(V\left(T_{2,i},M_{0,i}\right)\right)\right\|}_{2}$ and ${\left\|\nabla_{M_{0}}\left(V\left(T_{2,i},M_{0,i}\right)\right)\right\|}_{2}$.

Then solving Equation (3) also finds a (local) minima of Equation (4) if ${\left\|\nabla_{T_{2}}\left(V\left(T_{2,i},M_{0,i}\right)\right)\right\|}_{2}$ and ${\left\|\nabla_{M_{0}}\left(V\left(T_{2,i},M_{0,i}\right)\right)\right\|}_{2}$ approach 0 as the proposed framework’s iteration count, i, increases. We also take the final solution from the proposed framework, $\beta_{*}$ and $M_{0,*}$, estimate a T_2_ map, $T_{2,*}$, and solve the simulation-based reconstruction problem in Equation (4) using $T_{2,*}$ and $M_{0,*}$ as initialization. If the proposed framework appropriately minimizes Equation (4), then the solution will not change significantly from the initial guess. Finally, we compare computation times in optimizing the latent signal model- and simulation-based frameworks.

We implement G with a PyTorch EPG algorithm^58^ that enables computation of gradients with auto-differentiation to solve the EPG simulation–based reconstruction problem.

#### 2.4.6 | Prospective in vivo reconstruction experiments

We implemented a 2D-FSE sequence that enables random sampling of phase-encode lines in each echo of the echo train. For evaluation of latent signal models on prospectively undersampled datasets, our 2D-FSE sequence acquired data with four shots, random phase-encode ordering, 80 echoes, 5.56 ms echo spacing, 160 degree refocusing pulses, 256 × 256 mm FOV, 1 × 1 mm in-plane resolution, 3 mm slice thickness, and a 32-channel head coil. Because a time-resolved reconstruction aims to resolve 256 * 256 * 80 unknowns, this acquisition results in an overall prospective acceleration factor of $R=\frac{256\times 256\times 80}{256\times 80\times 4}=64$. The experiment compares linear subspace reconstructions with {4,6,8} degrees of freedom to the proposed latent signal model reconstruction with 3 degrees of freedom, both with no regularization. Auto-differentiation and BART solved the proposed and linear reconstruction problems, respectively.

### 2.5 | GE-EPTI experiments

Here, we demonstrate application of latent signal models in 2D GE-EPTI,^2,59^ which continuously measures k-space during $T_{2}^{*}$ dominated signal decay after an initial 90-degree excitation pulse. GE-EPTI resolves signal dynamics from the highly undersampled k_x_-k_y_-t dataset through B_0_-informed linear subspace reconstructions.

A fully sampled spatial and temporal multi-echo GE-EPTI dataset with matrix size = 216 × 216, slice thickness = 3 mm, 1.1 × 1.1 mm in-plane resolution, echo spacing = .93 ms, T=40 echoes, and 32 coils was acquired. To produce a challenging case, the undersampling mask modeled a GE-EPTI acquisition with two shots. This corresponds to 80 sampled k-space lines, resulting in an overall acceleration factor of $R\;=\;\frac{216*40}{80}\;=\;108$.

The first experiment generated from the fully sampled dataset. Different from T_2_-shuffling and MPRAGE, EPTI models the temporally varying phase, so both the linear and latent signal model reconstructions might be more sensitive to bias in B_0_ phase estimation in this highly undersampled experiment. We started with more accurate phase estimates to evaluate reconstructions independent of the B_0_-estimation algorithm.

We then repeated the experiment using a B_0_ map estimated from the central 49 k-space lines of the first six echoes to characterize performance with low-resolution phase. These central k-space lines were treated as a calibration prescan and not among the 80 lines used for reconstruction.

The experiments compare linear subspace reconstructions with {4,6} degrees of freedom and locally low-rank regularization to the proposed reconstruction with 3 degrees of freedom (1 latent variable) and wavelet regularization. The proposed approach trained an auto-encoder with two fully connected layers for both the encoder and decoder with tanh activations on a dictionary of simulated GE-EPTI signal evolution with $T_{2}^{*}$ values in the range of 10–300 ms. We chose all experimentally optimized regularization parameters to minimize error.

### 2.6 | MPRAGE-shuffling experiments

MPRAGE-shuffling^7^ employs linear subspaces to resolve multiple image contrasts across the MPRAGE echo train. We apply the proposed framework to model MPRAGE signal and reconstruct undersampled MPRAGE-shuffling data.

#### 2.6.1 | MPRAGE dictionary compression

First, we compare how efficiently SVD-generated linear subspaces and a trained auto-encoder represent single-tissue MPRAGE signal evolution. Bloch simulations generated training and testing dictionaries of MPRAGE signal evolution for T_1_ = 500–3000 ms, 256 echoes (T=256), 8 degree flip angle, and 7.8 ms echo spacing. Linear subspaces with {1,2,3,4} singular vectors (resulting in reconstruction problems with {2,4,6,8} degrees of freedom per voxel) and auto-encoders with {1,2,3,4} latent variables (resulting in reconstruction problems with {3,4,5,6} degrees of freedom per voxel) were generated from the training set. Each auto-encoder utilized two fully connected layers for both the decoder and encoder with hyperbolic tangent (tanh) activation functions. Resultant compression accuracies in the testing dictionary were compared.

#### 2.6.2 | MPRAGE reconstruction experiments

Prospectively undersampled MPRAGE-shuffling data was acquired using a spatial matrix size of 256 × 256 × 256, 1 mm isotropic resolution, 32-channel head coil, turbo factor (echo train length) of 256, 1100 ms TI, 2500 ms TR, 7.8 ms echo spacing, and 648 s total acquisition time. The 648 s acquisition time corresponds to a fully sampled non–time-resolved MPRAGE acquisition, but the MPRAGE-shuffling sequence distributed the phase/partition encode points randomly throughout the echo train. 16-channel SVD coil-compression^60^ and a read-out Fourier transform were applied to process an axial slice for the experiment. The data were further undersampled by excluding all k-space points measured after 324 s, resulting in an overall acceleration factor of $R\;=\;\frac{256*256*256}{256*128}\;=\;512$.

The reconstruction experiment compares linear subspace–constrained reconstructions with 4 degrees of freedom and wavelet or locally low-rank regularization to the latent signal model reconstruction with 3 degrees of freedom, wavelet regularization, and the MPRAGE trained auto-encoder.

All reconstructions were performed with varying regularization levels, and the best qualitative images are displayed. Auto-differentiation in PyTorch and BART solved the proposed and linear problems, respectively.

### 2.7 | Experiments to characterize the effects of partial volume voxels

Voxels at tissue boundaries produce linear combination of signals from multiple tissues present in the voxel. Whereas linear subspace techniques implicitly model this effect, because linear combinations of signals remain in the subspace, nonlinear models do not guarantee similar performance. We perform three sets of experiments to evaluate latent signal models in the presence of partial volumes: (1) evaluating how well linear subspaces and latent signal model auto-encoders represent in- and out-of-phase linear combinations of CSF, white matter, and gray matter signal; (2) reconstruction of simulated fully sampled and undersampled data with partial volume effects; and (3) comparing reconstructed signals at CSF/gray matter tissue boundaries in prospectively undersampled MPRAGE-shuffling experiments. The Partial Volume section of Supporting Information (Figures S8–S14) provides further experimental details.

## 3 | RESULTS

### 3.1 | T_2_-shuffling experiments

#### 3.1.1 | Dictionary compression

**FIGURE 2:**
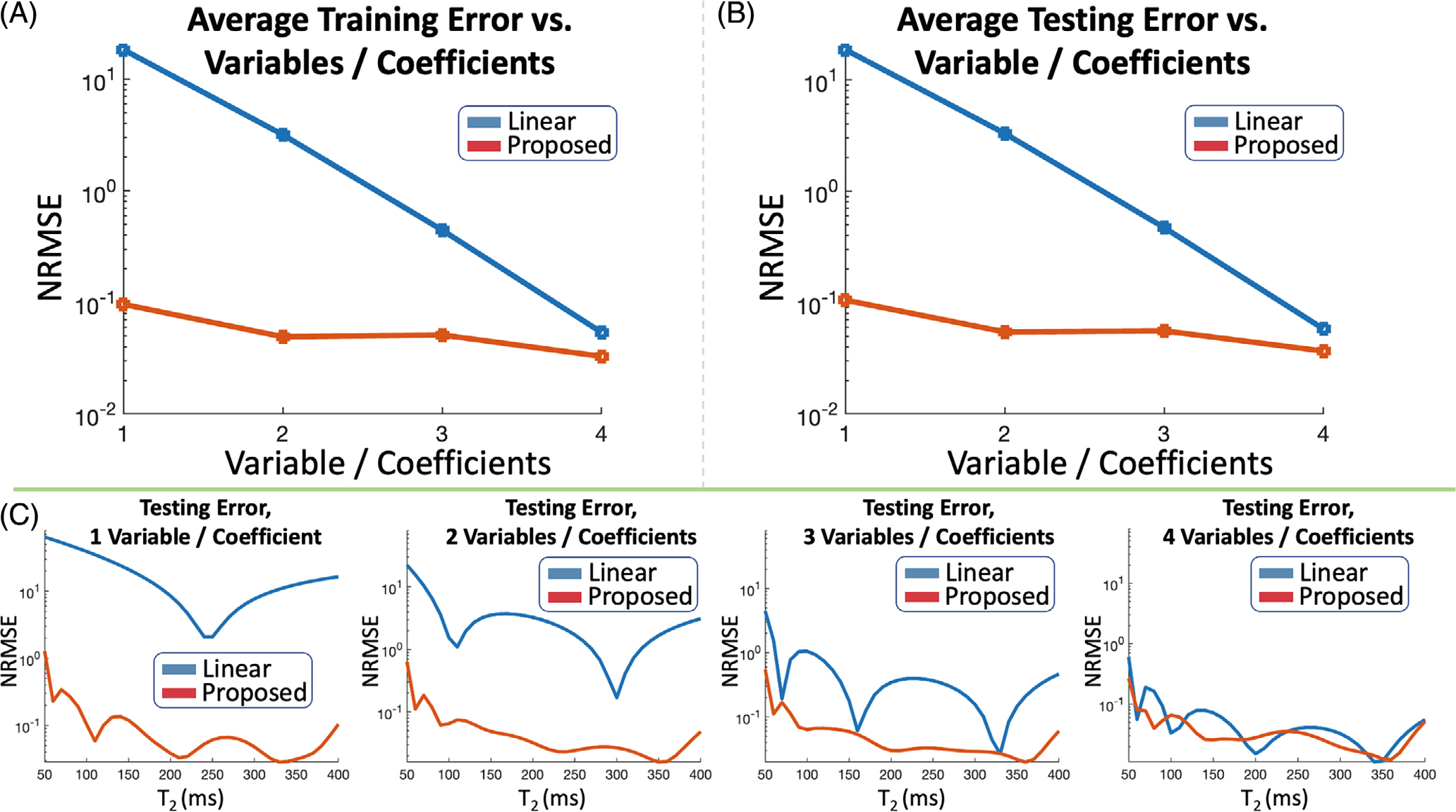
(A) + (B) Average error across the entire training and testing dictionaries of single-tissue FSE signal evolution using linear subspaces and proposed auto-encoders with {1,2,3,4} coefficients or latent variables. (C) Error for individual signal evolutions, associated with different T_2_ values, in the testing dictionary using subspaces and auto-encoders. The subspace requires 3–4 coefficients (resulting in {6,8} degrees of freedom per voxel during reconstruction), whereas the auto-encoder effectively captures signal evolution with just 1 latent variable (resulting in 3 degrees of freedom per voxel during reconstruction)

**FIGURE 3:**
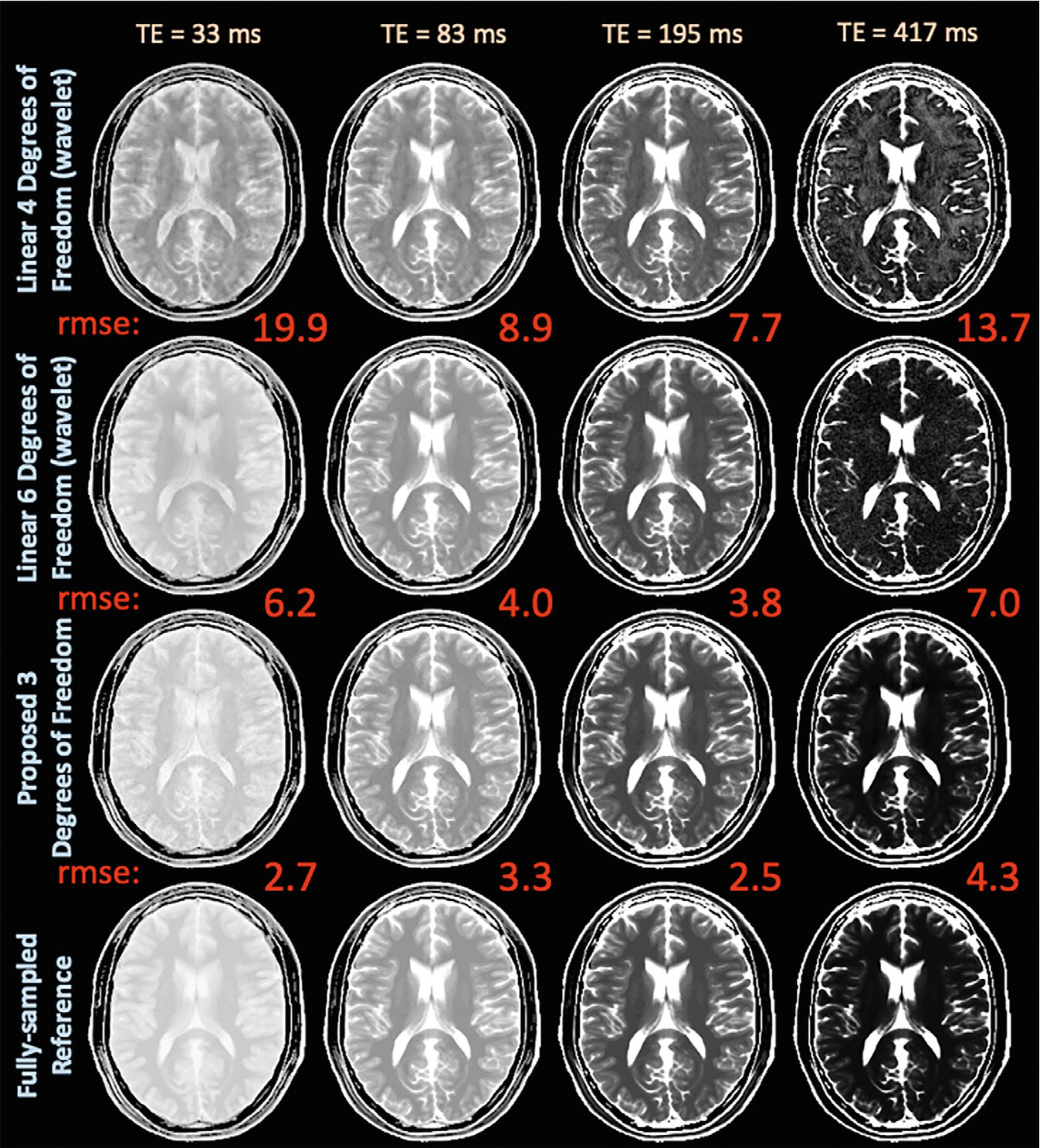
Exemplar reconstructed echo images and associated NRMSE from a simulated T_2_-shuffling acquisition comparing the subspace and proposed latent signal model reconstruction with {4,6} and 3 degrees of freedom, respectively. The reduced degrees of freedom enabled by inserting the decoder into the T_2_-shuffling forward model yields cleaner images and lower NRMSE. NRMSE, normalized RMS error.

In Figure 2A,B, the proposed auto-encoder reconstructs the single-tissue FSE signal training dictionary with {0.10%, 0.05%, 0.05%, 0.03%} average NRMSE and the testing dictionary with {0.11%, 0.05%, 0.06%, 0.04%} NRMSE with {1,2,3,4} latent variables (which produce reconstructions with {3,4,5,6} degrees of freedom). Linear subspaces with {1,2,3,4} coefficients (which produce reconstructions with {2,4,6,8} degrees of freedom) yield {18.41%, 3.18%, 0.44%, 0.05%} and {18.68%, 3.30%, 0.47%, 0.06%} NRMSE on the training and testing dictionaries. Figure 3C shows plots of NRMSE versus T_2_ value on individual signal-evolution entries from the testing dictionary.

#### 3.1.2 | Simulated reconstruction experiments

Simulation experiments in Figure 3 compare the proposed latent signal model reconstruction with 3 degrees of freedom per voxel and wavelet regularization to the linear reconstructions {4,6,8} degrees of freedom per voxel and wavelet or locally low-rank regularization on the *simulated_t2shfl1* dataset. Selected echo images and error maps in Figure S1A demonstrate that linear reconstructions with 4 degrees of freedom cannot adequately represent signal, whereas 6 linear degrees of freedom exhibit increased noise amplification. The proposed approach accurately represents signals and improves reconstruction performance. In Figure S1B, the proposed approach achieves lower NRMSE across all echoes. Linear wavelet with {4,6} degrees of freedom achieves {9.6,4.6}% average NRMSE across all echoes, whereas the proposed approach achieves 3.2%.

#### 3.1.3 | Retrospective in vivo reconstruction experiments

**FIGURE 4:**
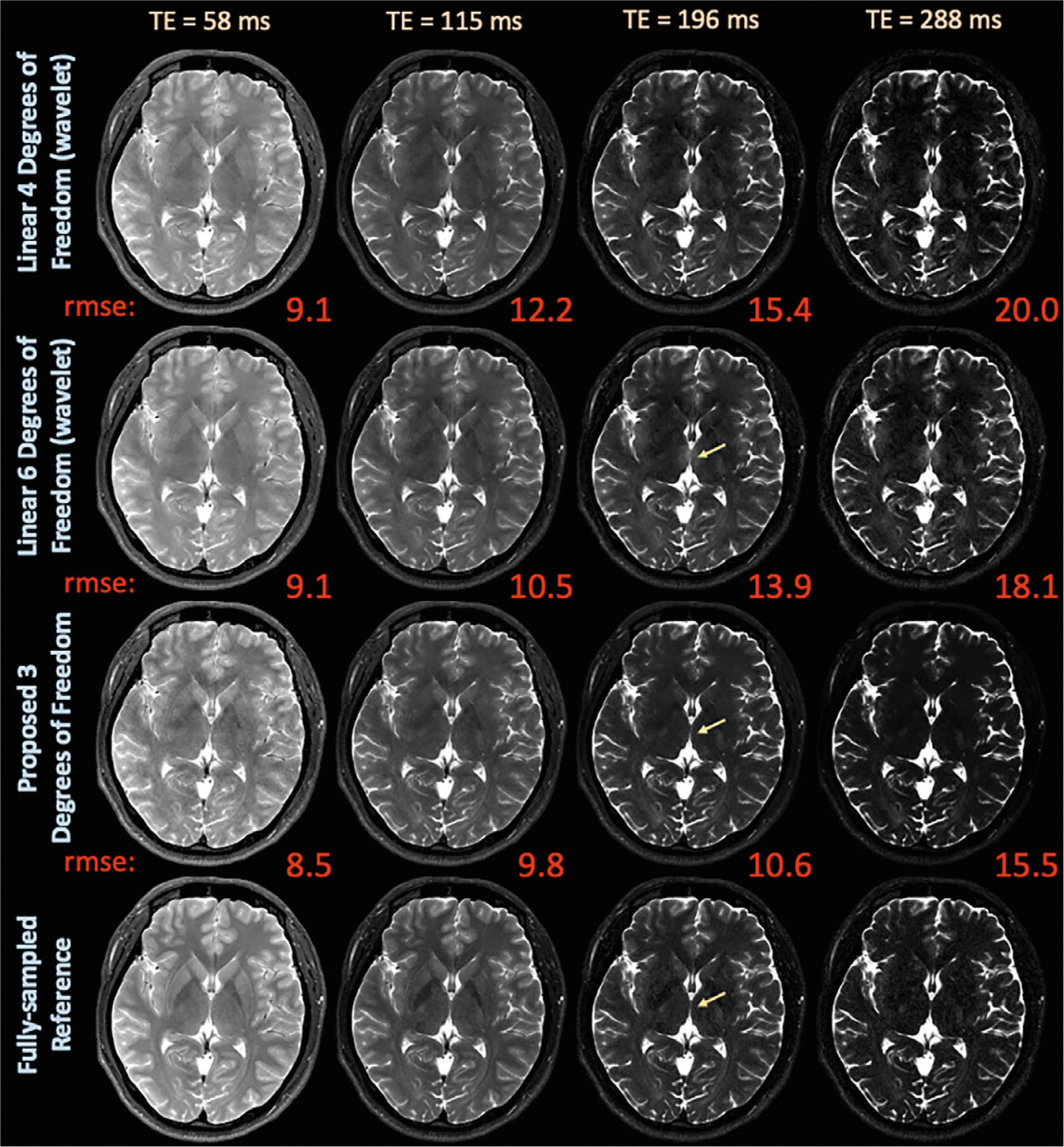
Exemplar reconstructed echo images and associated NRMSE maps from the in vivo, seven-shot retrospective; 2D–T_2_-shuffling acquisition at exemplar TEs comparing subspace constraints with {4,6}; and the proposed approach with 3 degrees of freedom. Like in simulation, the proposed approach yields cleaner images and lower NRMSE through reduced degrees of freedom

In vivo, retrospectively undersampled experiments in Figure 4 compare the proposed latent signal model reconstruction with 3 degrees of freedom per voxel and wavelet regularization to the standard linear reconstruction with {4,6,8} degrees of freedom per voxel with wavelet or locally low-rank regularization on the *invivo_t2shfl1 dataset*. (A) Selected echo reconstructions and error maps in Figure S2A show that the proposed framework reduces artifacts. More so, the proposed technique achieves lower NRMSE, as shown in Figure S2B, with average NRMSE across all echoes of 13.8% in comparison to {15.8%, 16.8%} and {16.8%, 15.2%} of the linear reconstructions.

Figure S3 presents a hyperparameter ablation experiment for the auto-encoder applied to retrospectively undersampled eight shots of this T_2_-weighted in vivo dataset. Informed by the results, we used the hyperbolic tangent nonlinearity with either two or three fully connected layers in the encoder and decoder for all applications.

#### 3.1.4 | Reconstruction stability across various noise instances

**FIGURE 5:**
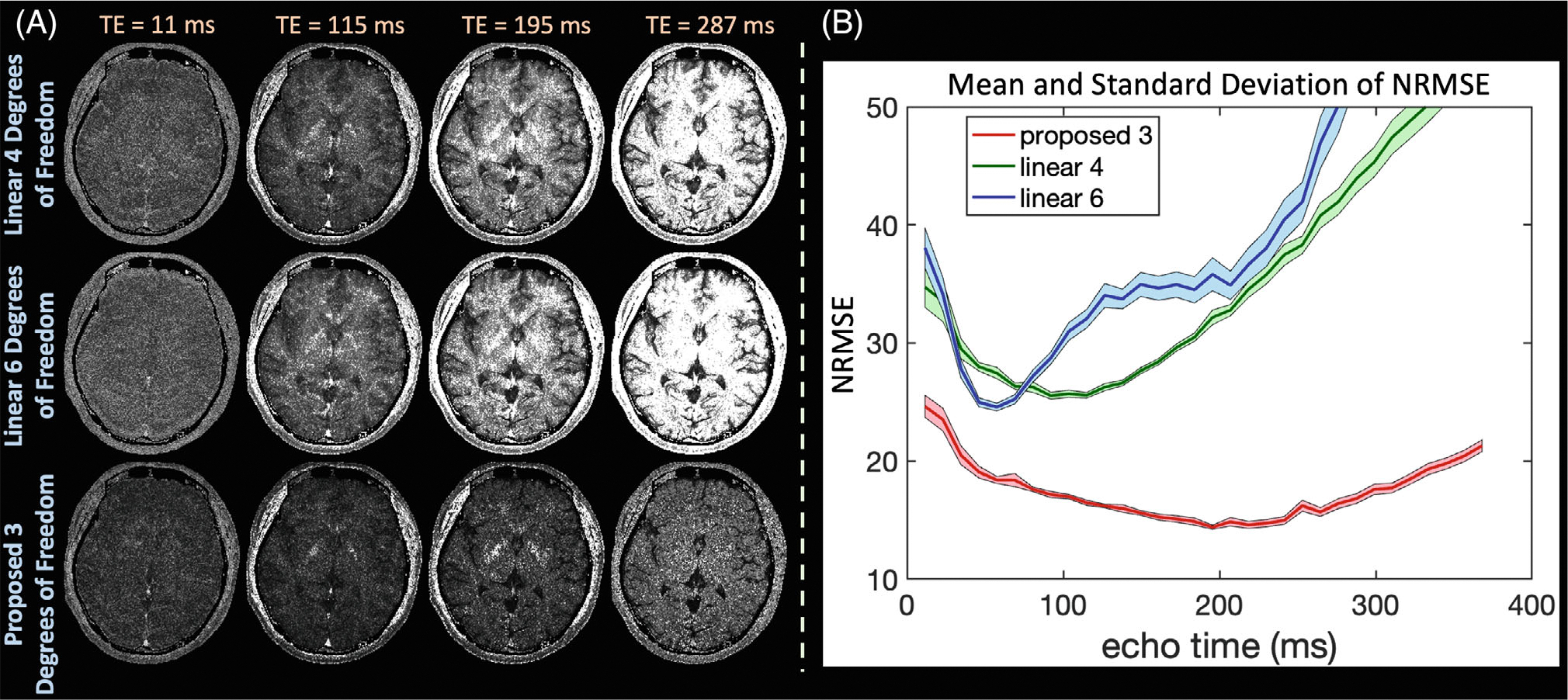
(A) Exemplar average absolute error maps for the proposed and linear reconstructions across the 250 k-space instances in the retrospective in vivo dataset. (B) Average and SD of reconstruction NRMSE at each echo. With its reduced degrees of freedom, the proposed latent signal model approach achieves lower average error while maintaining similar variance across the k-space instances in comparison to reconstructions with linear subspace constraints

Figures 5 and S4 analyzes linear and proposed reconstructions without regularization across the 250 different k-space instances in in vivo and simulation experiments, respectively. The proposed approach yields lower average absolute error maps in comparison to the linear reconstructions. Additionally, the proposed approach achieves lower average NRMSE across all echoes and maintains comparable variance in reconstruction accuracy.

#### 3.1.5 | Learning a proxy for differentiating through MRI simulations in a reconstruction setting

**FIGURE 6:**
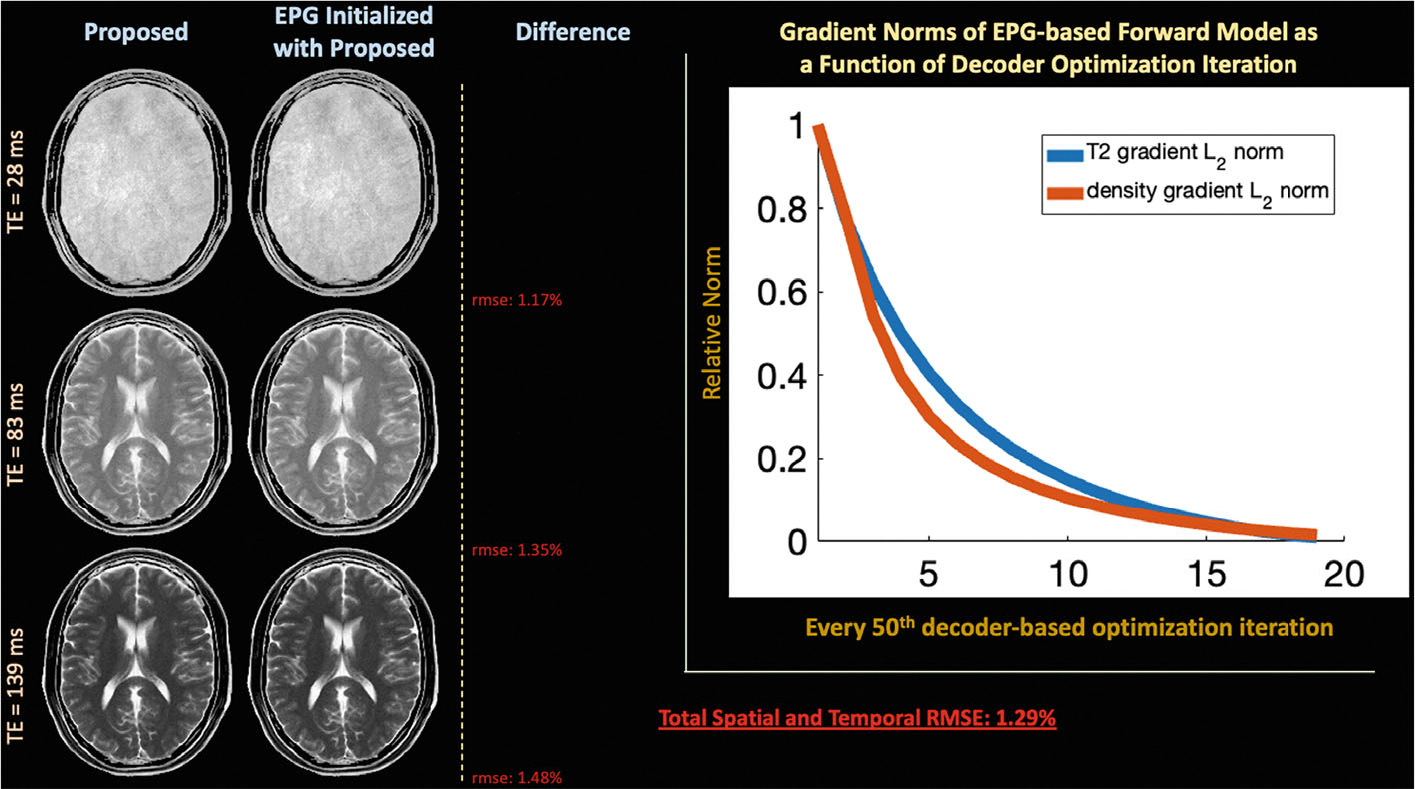
Plots gradient norms with respect to T_2_ and $M_{0}$ of the EPG-based forward model as a function of the latent signal model optimization iteration on the simulated dataset and displays exemplar reconstructed echo images from the proposed approach and the EPG-based forward model initialized with the proposed approach. The gradient norms approaching 1% of the maximum value and the quantitative similarity in the reconstructions suggest that the proposed approach efficiently finds a solution to the EPG-based forward model EPG, extended phase graph.

Figures 6B and S5B plots norms of gradients with respect to T_2_ and $M_{0}$ in the EPG-based forward model from Equation (4) as a function of every 50th latent signal model optimization iteration in simulation and in vivo, respectively. The norm of the gradients approach 1.1% and 2.7% of the maximum value in the simulation and in vivo retrospective experiments, respectively. Figures 6A and S5A display the proposed reconstruction, the EPG-based forward model reconstruction when initialized with T_2_ and $M_{0}$ estimates from the proposed reconstruction, and their absolute difference. The absolute error maps and the total spatial–temporal NRMSE of less than 1.3% between the two techniques suggests that the proposed optimization framework finds a solution for the EPG-based forward model.

#### 3.1.6 | Prospective in vivo reconstruction experiments

**FIGURE 7:**
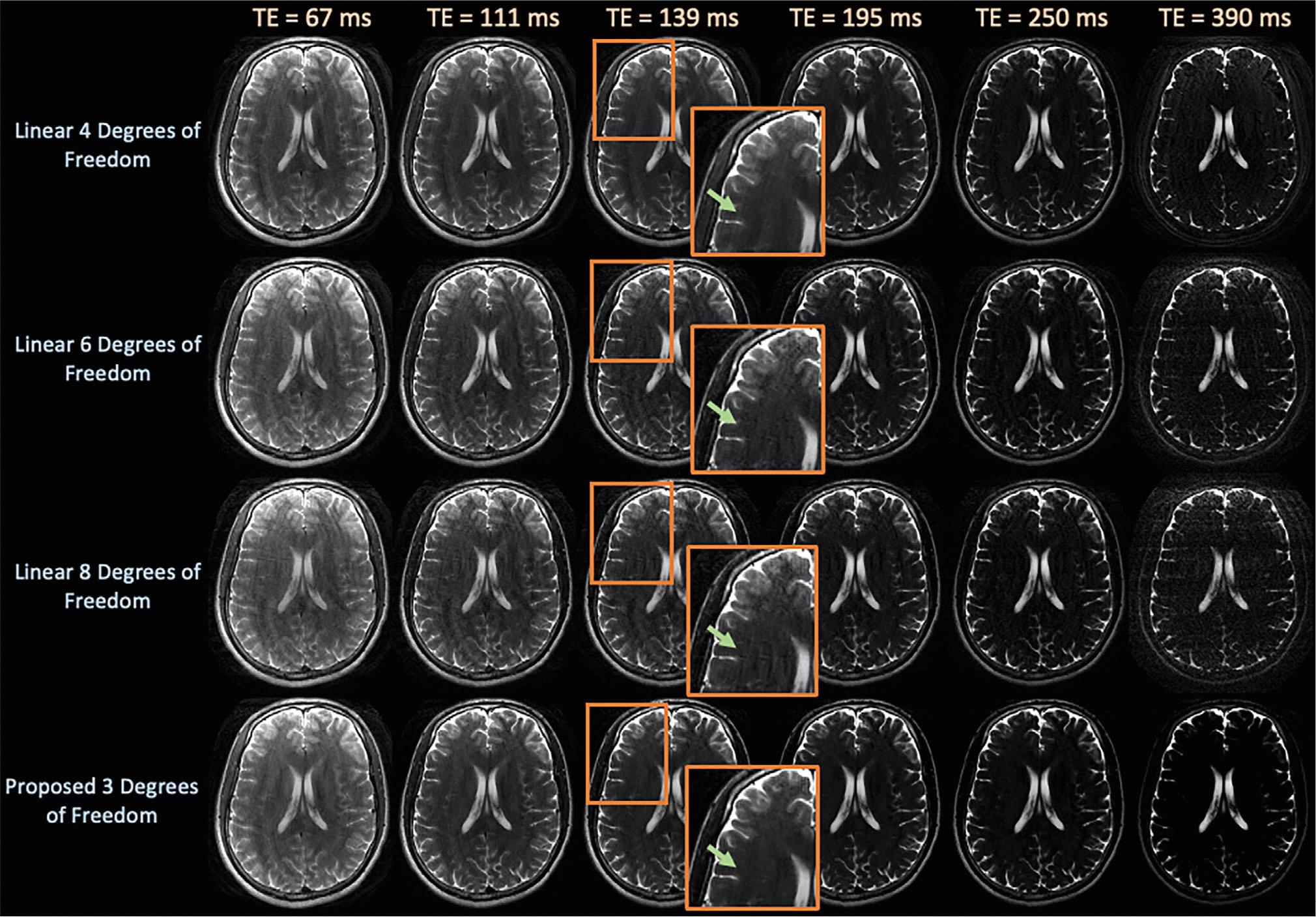
Images from an in vivo, prospective 2D-shuffled–FSE acquisition with four shots at exemplar TEs comparing linear subspace reconstructions with {4,6,8} and the proposed approach with 3 degrees of freedom. The proposed technique yields qualitatively cleaner images, as highlighted by the zoomed images and arrows, by employing a forward model with fewer degrees of freedom in comparison to the linear approach

Figure 7 displays latent signal model and linear subspace reconstructions at exemplar TEs on the prospectively acquired 2D-FSE dataset. The proposed technique yields reduced reconstruction artifacts, particularly at the later 390 ms TEs.

#### 3.1.7 | Comparison of total reconstruction times

With a 32 GB T V100 GPU (NVIDIA, Santa Clara, California), total reconstruction times for 1000 iterations on the simulated dataset were 7.5 s for linear subspaces with 8 degrees of freedom, 2509.3 s for the EPG-based forward model, and 93.6 s for the proposed latent signal model approach. Analogous reconstruction times on the in vivo dataset were 4.62 s for linear subspaces with 8 degrees of freedom, 448.4 s for the EPG-based forward model, and 62.2 s for the proposed approach.

### 3.2 | GE-EPTI experiments

**FIGURE 8:**
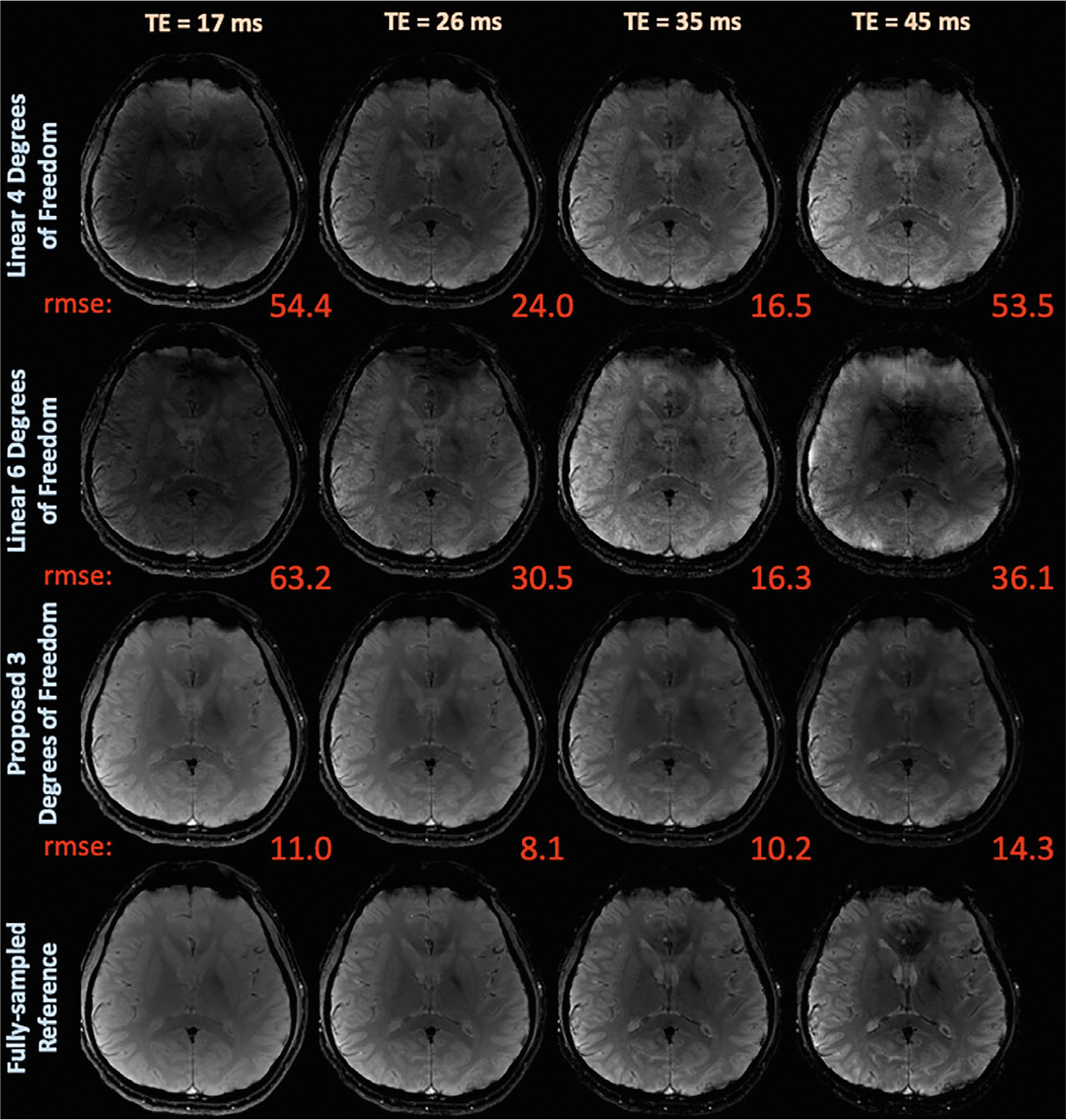
Exemplar reconstructed echo images and associated NRMSE from the GE-EPTI dataset using phase estimated from the fully sampled dataset. The proposed latent signal model approach with reduced degrees significantly improves qualitative image quality and quantitative NRMSE in comparison to the linear subspace constrained reconstructions EPTI, echo-planar-time-resolved-imaging; GE, gradient echo.

Figure 8A compares exemplar reconstructed echo images, and Figure S6A compares error maps using linear subspaces with locally low-rank regularization and the latent signal models with wavelet regularization from the in vivo GE-EPTI dataset with phase estimated from fully sampled data.

In this challenging case with high undersampling, the linear reconstructions suffer from bias and artifacts, whereas the proposed approach significantly improves reconstruction quality. Figure S6b shows that the proposed approach yields quantitative improvements, with average NRMSE across all echoes of 11.48%, in comparison to the linear average errors of {38.0%, 40.3%}.

Figure S7 displays the same reconstructions from Figure 8 with phase estimates calibrated from low-resolution data. Linear reconstructions remain poor, whereas the proposed approach still achieves significant relative improvement.

### 3.3 | MPRAGE-shuffling experiments

#### 3.3.1 | MPRAGE dictionary compression

**FIGURE 9:**
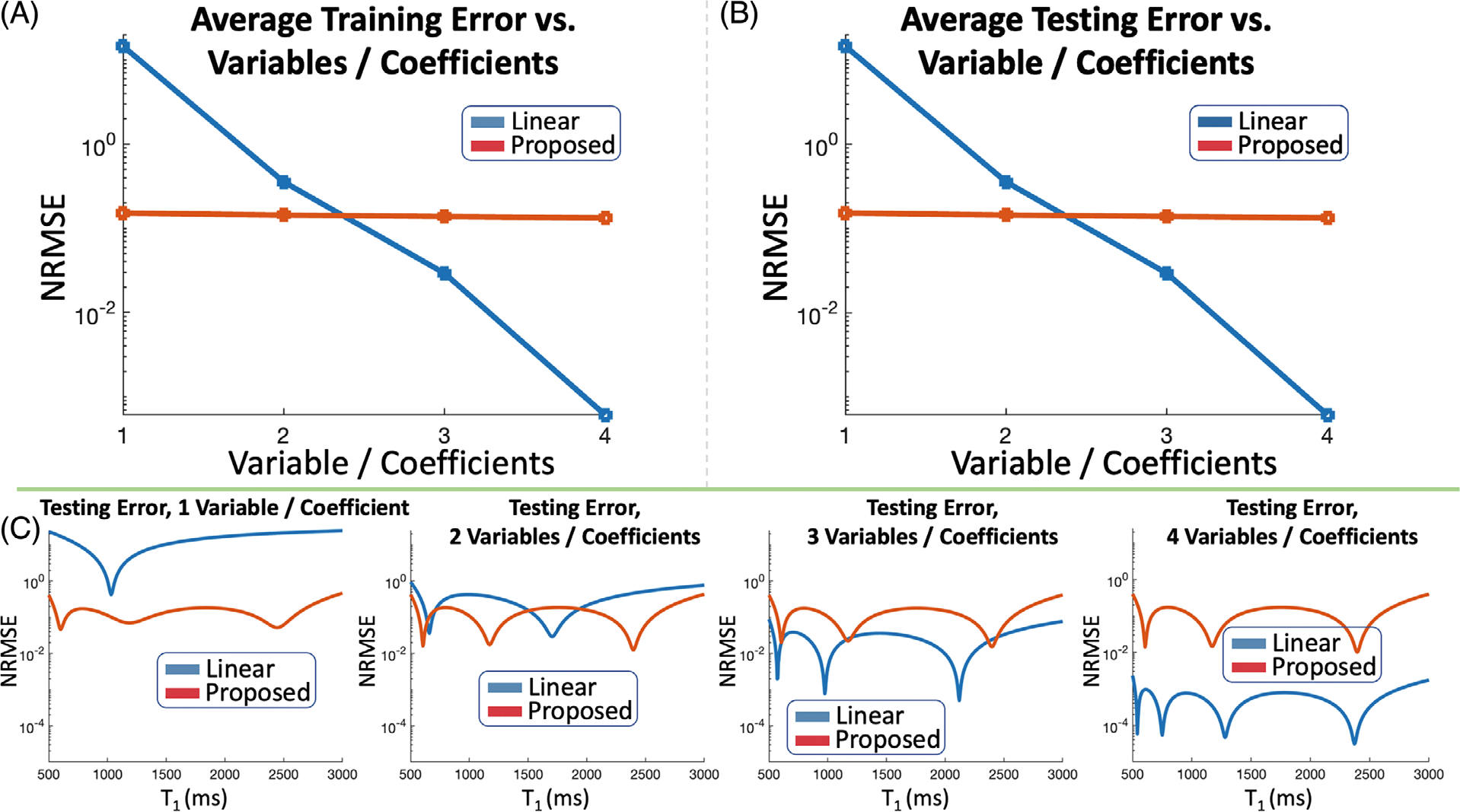
(A) + (B) Average error across the entire training and testing dictionaries of MPRAGE signal evolution using linear subspaces and proposed auto-encoders with {1,2,3,4} coefficients or latent variables. (C) Error for individual signal evolutions, associated with different T_1_ values, in the testing dictionary using subspaces and auto-encoders. The subspace requires 2–3 coefficients (resulting in {4,6} degrees of freedom per voxel during reconstruction), whereas the auto-encoder effectively captures signal evolution with just 1 latent variable (resulting in 3 degrees of freedom per voxel during reconstruction)

In Figure 9A,B, the proposed auto-encoder reconstructs the single-tissue MPRAGE signal training dictionary with {0.15%, 0.14%, 0.14%, 0.13%} average NRMSE and the testing dictionary with {0.15%, 0.14%, 0.14%, 0.13%} NRMSE with {1,2,3,4} latent variables, respectively (which produce reconstructions with {3,4,5,6} degrees of freedom). Linear subspaces with {1,2,3,4} coefficients (which produce reconstructions with {2,4,6,8} degrees of freedom) yield {14.5%, 0.36%, 0.03%, 6.0 × 10^−4^%} NRMSE and {15.0%, 0.36%, 0.03%, 6.0 × 10^−4^%} NRMSE on the training and testing dictionaries, respectively. Figure 3C shows plots of NRMSE versus T_1_ value on individual signal-evolution entries from the testing dictionary.

#### 3.3.2 | MPRAGE reconstruction experiments

**FIGURE 10:**
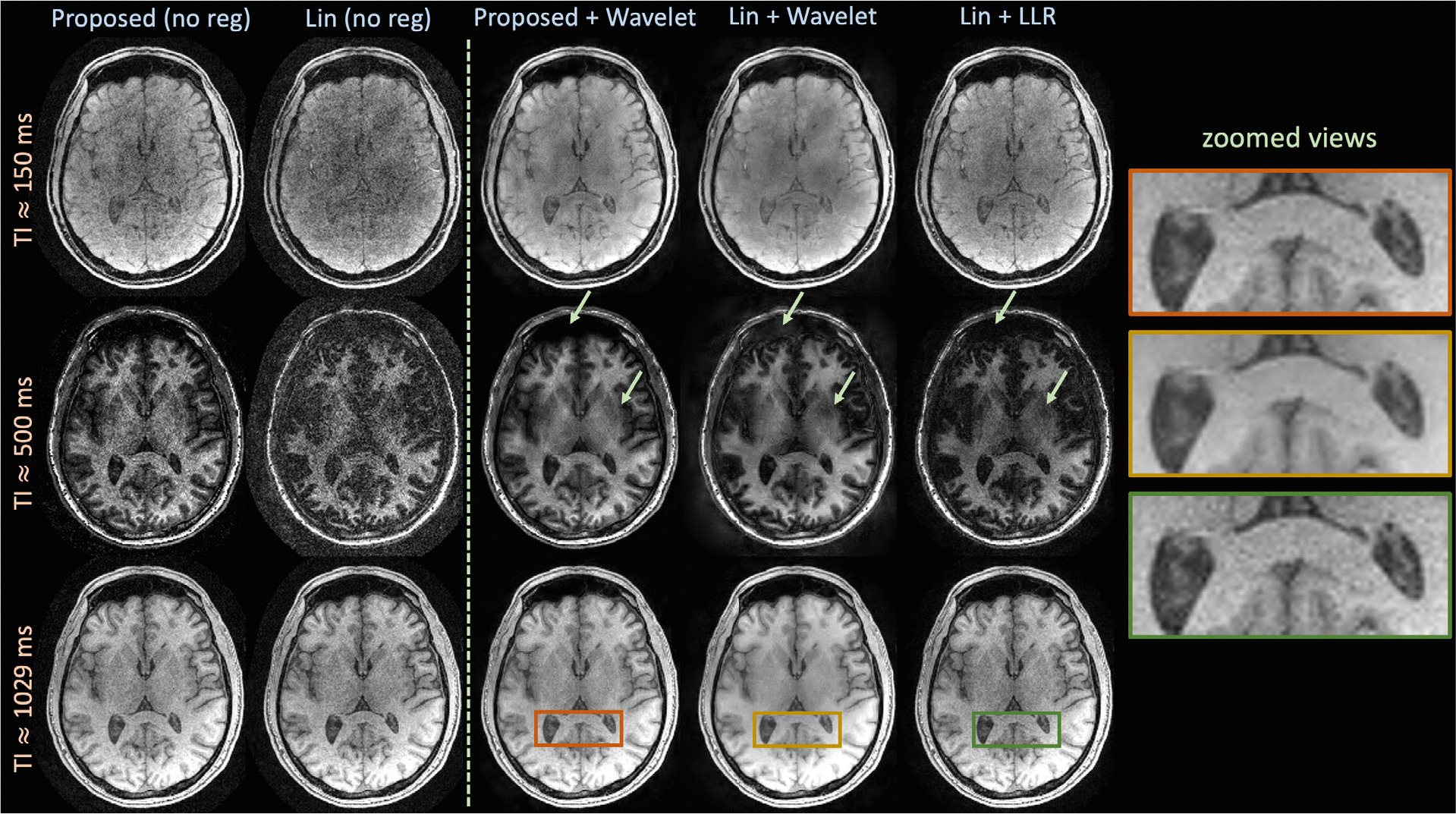
Reconstructions with and without regularization on the MPRAGE-shuffling dataset comparing the proposed latent signal model framework and linear subspace constraints. Without regularization, the proposed approach significantly reduces noise amplification and improves reconstruction quality. With applied regularization, linear + wavelet suffers increased blurring, whereas linear + locally low rank still exhibits noise amplification. The proposed approach reduces reconstruction artifacts, particularly at TI ≈ 500 ms, improves image sharpness in comparison to linear + wavelet, and reduces noise amplification in comparison to linear + locally low rank

Figure 10 displays reconstructions of the MPRAGE-shuffling dataset. The first two columns compare un-regularized reconstructions from the proposed and linear approaches, with 3 and 4 degrees of freedom, respectively. The proposed latent signal model approach significantly reduces apparent noise amplification at the displayed inversion times (3 out of 256). The third, fourth, and fifth columns display proposed with wavelet regularization and linear with wavelet or locally low-rank regularization, respectively. Particularly at TI ≈ 500 ms, the proposed approach reduces reconstruction artifacts in comparison to the linear techniques. The zoomed views suggest that the wavelet-regularized linear reconstruction reduces noise amplification at the cost of significant blurring in the image, whereas the locally low-rank linear reconstruction suffers from noise amplification. The regularized latent signal model reconstruction reduces artifacts and noise while maintaining sharpness.

### 3.4 | Experiments to characterize the effects of partial volume voxels

Figures S8–S11 compare performance of latent signal models and linear subspaces representing in-phase additive and out-of-phase subtractive FSE and MPRAGE partial volume signals. Whereas subspaces with 4 real coefficients represent partial volumes the best, latent signal models represent additive FSE signal within 4% NRMSE, additive MPRAGE signal within 0.5% NRMSE, and subtractive MPRAGE signal within 3.1% NRMSE. However, latent signal model represents subtractive FSE signal poorly.

Figures S12 and S13 compare reconstructions on fully sampled and undersampled FSE and MPRAGE data with partial volume effects. Whereas linear models achieve improved performance with fully sampled data, latent signal models achieve better performance with undersampled data.

Figure S14 shows that signal phase and magnitude at CSF/gray matter boundary voxels from subspace and latent signal model reconstructions of MPRAGE-shuffling follow similar trajectories.

## 4 | DISCUSSION

Latent signal models improve time-resolved, multi-contrast MRI reconstruction by combining ideas from Bloch equation–based techniques, linear subspace constraints, and latent representation of signals. By training an auto-encoder on dictionaries of signal evolution, our technique learns compact, latent representations of single-tissue signals. Then, inserting the decoder into the forward model reduces degrees of freedom and serves as a proxy for fast and feasible optimization through the Bloch equations. Our proposed method builds upon previous work that utilizes auto-encoders to regularize diffusion^44,45^ and MRSI^44,45^ by extending applications to multi-contrast imaging and incorporating the decoder portion of the auto-encoder directly in the forward model, obviating the separate regularization term. Latent signal models represent single-tissue FSE, EPTI, and MPRAGE signal evolution with 1 latent variable to within 0.15% average NRMSE and improve reconstruction quality in undersampled single-tissue simulated T_2_-shuffling, in vivo T_2_-shuffling, in vivo GE-EPTI, and in vivo MPRAGE-shuffling acquisitions with 3 degrees of freedom per voxel, in comparison to linear subspace–constrained reconstructions with {4,6,8} degrees of freedom.

In the settings explored, signal largely depends on a single underlying tissue parameter (T_2_ for FSE, $T_{2}^{*}$ for GE-EPTI, T_1_ for MPRAGE-shuffling), so the auto-encoder essentially learns a one-to-one 1D transformation between the latent variable and underlying tissue parameter as shown in Figure S15. However, our technique does not explicitly build this knowledge into the learning algorithm; rather, the model implicitly learns to compress signal into 1 latent variable from the dictionary of simulated signal evolution.

Recently, “unrolled” reconstructions utilizing neural networks as learned regularization achieve state-of-the-art results for structural image reconstruction.^61^ However, impracticality of acquiring fully sampled data precludes widespread use of these techniques in time-resolved applications. Because we simulate the dictionary, the proposed latent signal model framework does not require acquisition of any fully sampled datasets for model training. In addition, the auto-encoder is trained once and can be reused with subsequent acquisitions from the same sequence. Finally, incorporating the proposed framework directly in the forward model obviates the selection of a regularization parameter, “lambda.” Some recent work incorporates subspace models into the data-consistency term of unrolled reconstructions;^62^ thus, as future directions, we envision combining latent signal models with subspace unrolled reconstructions by replacing the subspace model in the data-consistency block with a decoder when appropriate data is available.

Most of the reconstruction experiments in this work employ spatial regularization in the proposed and linear reconstructions for fair comparisons. We included Figure 5 to isolate the effects from reduced degrees of freedom and understand our technique’s sensitivity to different noise instances. As the figure suggests, the reduced degrees of freedom provide reliable improvements in reconstruction quality. Combining the proposed latent signal model approach with spatial regularization then yields additive improvements, as demonstrated in the other experiments.

In the T_2_-shuffling setting, Figure 6 demonstrated that the proposed technique finds local minima of reconstruction problems that incorporate EPG-simulations in the forward model. Our EPG-based comparisons implemented auto-differentiation through EPG simulations^58,61^ to calculate gradients for reconstruction. In comparison to auto-differentiating through the EPG simulations, our proposed approach reduced reconstruction time by at least an order of magnitude. Thus, we envision our approach being advantageous when differentiation through the signal simulation requires significant computation, like isochromat-based simulations.^28,62^ On the other hand, the latent signal model auto-encoders need to be retrained for varying acquisitions and sequence parameters, whereas Bloch equation–based auto-differentiation only requires a differentiable implementation of new sequences of interest.

Latent signal models improved significantly utilizing a fully sampled phase estimate in comparison to a low-resolution estimate when reconstructing EPTI data in Figures 8 and S7. Thus, future work will explore improved B_0_ estimation and refinement^48,63,64^ techniques to improve phase estimates for latent signal model EPTI reconstructions.

Linear subspace techniques, like T_2_-shuffling^6^ in FSE applications, improve image sharpness by modeling signal dynamics that typically modulate k-space and produce blurring in non–time-resolved images. Figure S16 demonstrates that latent signal models and linear subspaces yield similar improvements in image sharpness on a simulated T_2_-shuffling dataset. Further details can be found in the Deblurring Comparisons of Latent Signal Models and Linear Subspace Reconstructions section of Supporting Information (Supporting Information Figure S16).

Deep reconstruction network (DRONE),^65^ a technique for quantitative mapping, also trains neural networks to learn a function between tissue parameters and Bloch-simulated signal evolution. However, DRONE requires signal evolution that has already been reconstructed using some conventional technique as input to its network to estimate quantitative maps. On the other hand, latent signal models is a proposed time-resolved reconstruction technique that utilizes its neural networks directly in the imaging forward model to reconstruct signal evolution from acquired k-space data. Future research directions in quantitative mapping could combine both techniques by using DRONE to estimate quantitative tissue parameter maps from signal evolution reconstructed with latent signal models.

### 4.1 | Limitations

Our partial volume experiments suggest that linear models with 3–4 real coefficients represent mixtures of signal better than latent signal model and reconstruct higher quality images with fully sampled data. In addition, latent signal models break down in the presence of subtractive FSE partial volumes. However, in undersampled reconstruction experiments with partial volume effects (Figures 4, 8, S12, and S13), latent signal model still achieves better reconstruction metrics overall. We suspect this occurs because the data do not contain significant subtractive FSE signals, the reconstruction metrics evaluate error throughout the entire image, not just the partial volume voxels, and latent signal models represent partial volumes well enough while reducing noise amplification more effectively. Future work could involve incorporating partial volume examples into the training procedure of latent signal models.

The presence of neural networks in the proposed framework induces a nonconvex optimization problem that may converge to a suboptimal local minimum with an improperly trained auto-encoder. For example, the LeakyRelu autoencoders, explored in Figure S3, yield significant variability in performance from different random initialization of model weights. We found that tanh activations produce reconstruction problems that reliably terminate at reasonable local minima. Avoiding suboptimal local minima in other applications may require changes to the auto-encoder structure.

Whereas the proposed approach significantly improves reconstruction times in comparison to the EPG-based forward model, linear models reconstruct 10–12× faster because the Fourier and Coil Operators, *FS*, commutes with the temporal operator,^6^ ϕ, in Equation (1) when the phase operator is H=1, significantly reducing computation in the shuffling regime. However, FS does not commute with Q in Equation (3), so our technique required increased computational complexity in comparison to linear shuffling. Note, these operators do not commute when H≠1, so the computational advantages of linear reconstructions diminish in EPTI applications.

## 5 | CONCLUSION

With the proposed latent signal model framework, directly solving for learned nonlinear latent representations of signal improves time-resolved MRI reconstruction through reduced degrees of freedom. T_2_-shuffling, EPTI, and MPRAGE-shuffling applications demonstrate quantitative and qualitative benefits of the proposed technique in simulation and in vivo experiments.

## Supplementary Material

Supplementary material **Figure S1.** (A) Selected reconstruction error maps and (B) NRMSE across all echoes comparing the proposed approach and linear reconstructions with the simulated T_2_-shuffling dataset. Linear reconstructions with 4 degrees of freedom cannot adequately represent signal, while 6 linear degrees of freedom exhibit increased noise amplification. The proposed approach achieves less error at the echoes, and lower NRMSE at all time-points.
**Figure S2.** (A) Selected reconstruction error maps and (B) NRMSE across all echoes comparing the proposed approach and linear reconstructions on the retrospectively under-sampled, in vivo T_2_-shuffling dataset. The proposed framework reduces NRMSE and image artifacts in comparison to the linear reconstructions.
**Figure S3.** Comparing the performance of different auto-encoder model hyper-parameters in in vivo retrospective reconstructions. (A) and (B) display grids of average NRMSE across all echoes for models with LeakyRelu and Tanh for a range of layers, learning rates, and epochs. LeakyRelu achieves lowest NRMSE with 2 layers, 200 K epochs, and 1e-5 learning rate, while Hyperbolic tangent achieves its minimum with 3 layers, 100 K epochs, and 1e−4 learning rate. (C) Plots the performance of LeakyRelu and Tanh, with their respective best hyper-parameters, at 8 different random initializations. LeakyRelu varies significantly, while tanh yields consistent results.
**Figure S4.** Linear and proposed reconstructions without regularization across 250 different k-space instances on the simulated T_2_-shuffling dataset. The proposed approach achieves lower average absolute error maps and lower NRMSE across all the echoes, while maintaining comparable variance in reconstruction accuracy.
**Figure S5.** (B) plots gradient norms with respect to T_2_ and density of the EPG-based forward model as a function of the Latent Signal Model Optimization iteration on the retrospectively under-sampled in-vivo dataset. (A) displays exemplar reconstructed echo images from the proposed approach and the EPG-based forward model initialized with the proposed approach. The gradient norms approaching 2.7% of the maximum value and the quantitative similarity in the reconstructions suggest that the proposed approach efficiently finds a solution to the EPG-based forward model.
**Figure S6.** (A) Selected reconstruction error maps and (B) NRMSE across all echoes comparing the proposed approach and linear reconstructions on the retrospectively under-sampled EPTI dataset. The proposed framework reduces NRMSE and image artifacts in comparison to the linear reconstructions.
**Figure S7.** Similar comparisons of the proposed and linear subspace approach from Figure 7 using phase estimated from low-resolution calibration data in the GE-EPTI forward model. (A) displays exemplar echo images and associated error maps and (B) plots NRMSE for each echo. Even with a worse phase-estimate, the proposed approach still produces higher quality images with lower NRMSE in comparison to the linear techniques.
**Figure S8.** Plots of estimated signal evolution from simulated, in-phase additive FSE partial volumes and resultant magnitude error with respect to the ground truth. While linear subspaces with 3 and 4 real coefficients achieve best results, the proposed auto-encoder achieves less than 4% NRMSE for all partial volume linear combinations.
**Figure S9.** Plots of estimated signal evolution from simulated, out-of-phase subtractive FSE partial volumes and resultant magnitude error. Latent Signal Models represent signal poorly in comparison to linear subspaces with 3 and 4 real coefficients, which achieve less than 5% error. However, Latent Signal Models do not seem to exhibit worse performance at tissue boundaries in comparison to linear models in the in-vivo T_2_ weighted experiments shown in Figure 4 of the main document. We suspect that this is due to the refocusing pulses in FSE sequences that induce minimal phase-variation within a single voxel resulting in additive partial volume mixtures, which Latent Signal Models do a much better job of representing.
**Figure S10.** Plots of estimated signal evolution from simulated, in-phase additive MPRAGE partial volums and resultant magnitude error with respect to ground truth. All techniques represent signals within 0.5% NRMSE.
**Figure S11.** Plots of estimated signal evolution from simulated, out-of-phase subtractive MPRAGE partial volumes and resultant magnitude error. While the linear models achieve better performance, the proposed technique represents signal within 3.1% NRMSE.
**Figure S12.** (A) shows reconstructions of selected echo times and corresponding error from fully sampled FSE reconstructions with partial voluming. Linear 4 suffer from bias due to the inability of 2 complex coefficients to adequately represent signal evolution and achieves 11.50% NRMSE. Linear 6 and 8 achieve best performance with 1.44% and 0.55% NRMSE respectively and exhibit noise-like error maps. The proposed approach achieves 2.03% average NRMSE with higher error along tissue boundaries. (B) displays selected echo times and corresponding error from under-sampled FSE reconstructions with partial voluming. Images from Linear 4 still exhibits bias with 10.10% average NRMSE while Linear 8 suffers from noise amplification resulting in 16.20% average NRMSE. Linear 6 balances noise amplification and signal representation with 5.83% average NRMSE, but the proposed approach achieves best reconstruction with 4.14% average NRMSE.
**Figure S13.** (A) shows reconstructions of selected inversion times and corresponding errors from fully sampled MPRAGE-shuffling data with partial voluming. The three linear models and the proposed approach all achieve high fidelity reconstructions with 0.77%, 0.56%, 0.64%, and 0.86% NRMSE respectively. Linear models yield noise-like error maps, while the errors in Latent Signal Models concentrate near tissue boundaries. (B) displays selected inversion times and error from under-sampled MPRAGE reconstructions with partial voluming. Noise amplification in the linear models yields 10.15%, 16.70%, and 23.51% average NRMSE for 4, 6, and 8 degrees of freedom respectively. The proposed approach achieves less qualitative noise amplification and the lowest average NRMSE of 8.86%.
**Figure S14.** Selected inversion times from un-regularized linear subspace and Latent Signal Model reconstructions on prospectively under-sampled MPRAGE shuffling data and temporal signal evolution magnitude and phase from voxels on the gray matter / CSF boundary. Even with this less under-sampled acquisition, Latent Signal Models reduce noise amplification in comparison to linear reconstructions. Additionally, both the phase and magnitude of signal evolution in the partial volume voxels follow similar trajectories.
**Figure S15.** (A) plots the relationship between the learned latent variable, β versus T2, for an auto-encoder trained on FSE signal evolution, while (B) plots β versus T1 for MPRAGE signal evolution. Since our FSE and MPRAGE simulations vary as a function of one underlying parameter, the auto-encoder essentially learns a one-to-one 1D transformation between the latent variable and T1 or T2.
**Figure S16.** Ground truth, a non-time resolved reconstruction using center-out-ordering k-space sampling, and a linear and our proposed time resolved reconstructions at matching echo times using random sampling. All reconstruction sampled data from a FSE acquisition. The non-time-resolved image suffers from blurring due to FSE signal decay modulating k-space. On the other hand, both the linear and proposed time-resolved techniques yield sharper images by modeling and reconstructing signal dynamics.

SUPPORTING INFORMATION

Additional supporting information may be found in the online version of the article at the publisher’s website.

## Data Availability

Code and data used to generate Figures 2–6, 9 can be found in the following two links, respectively: https://github.com/YaminArefeen/latent_signal_models_mrm_2022, https://www.dropbox.com/sh/5upn7121m0dxvjp/AAD2liymnq4LhNKs85nUddJya?dl=0
