# Supplementary material for: Latent signal models: Learning compact representations of signal evolution for improved time-resolved, multi-contrast MRI
Source: Magn Reson Med. Author manuscript; Available in PMC 2025 Oct 26. (PMC12554331; doi:10.1002/mrm.29657)
Supplement: Supplementary material — Figure S1. (A) Selected reconstruction error maps and (B) NRMSE across all echoes comparing the proposed approach and linear reconstructions with the simulated T2-shuffling dataset. Linear reconstructions with 4 degrees of freedom cannot adequately represent signal, while 6 linear degrees of freedom exhibit increased noise amplification. The proposed approach achieves less error at the echoes, and lower NRMSE at all time-points. Figure S2. (A) Selected reconstruction error maps and (B) NRMSE across all echoes comparing the proposed approach and linear reconstructions on the retrospectively under-sampled, in vivo T2-shuffling dataset. The proposed framework reduces NRMSE and image artifacts in comparison to the linear reconstructions. Figure S3. Comparing the performance of different auto-encoder model hyper-parameters in in vivo retrospective reconstructions. (A) and (B) display grids of average NRMSE across all echoes for models with LeakyRelu and Tanh for a range of layers, learning rates, and epochs. LeakyRelu achieves lowest NRMSE with 2 layers, 200 K epochs, and 1e-5 learning rate, while Hyperbolic tangent achieves its minimum with 3 layers, 100 K epochs, and 1e−4 learning rate. (C) Plots the performance of LeakyRelu and Tanh, with their respective best hyper-parameters, at 8 different random initializations. LeakyRelu varies significantly, while tanh yields consistent results. Figure S4. Linear and proposed reconstructions without regularization across 250 different k-space instances on the simulated T2-shuffling dataset. The proposed approach achieves lower average absolute error maps and lower NRMSE across all the echoes, while maintaining comparable variance in reconstruction accuracy. Figure S5. (B) plots gradient norms with respect to T2 and density of the EPG-based forward model as a function of the Latent Signal Model Optimization iteration on the retrospectively under-sampled in-vivo dataset. (A) displays exemplar reconstructed echo images from the pro [file NIHMS2109892-supplement-Supplementary_material.pdf]

Supporting Information

The following supporting information accompanies the manuscript, “Latent Signal Models: Learning Compact Representations of Signal Evolution for Improved Time-Resolved, Multi-contrast MRI”.

Simulated Reconstruction Experiments Error Maps and RMSE Plot

Supporting Information Figure S1: (A) Selected reconstruction error maps and (B) RMSE across all echoes comparing the proposed approach and linear reconstructions with the simulated T_2-shuffling dataset. Linear reconstructions with 4 degrees of freedom cannot adequately represent signal, while 6 linear degrees of freedom exhibit increased noise amplification. The proposed approach achieves less error at the echoes, and lower RMSE at all time-points.

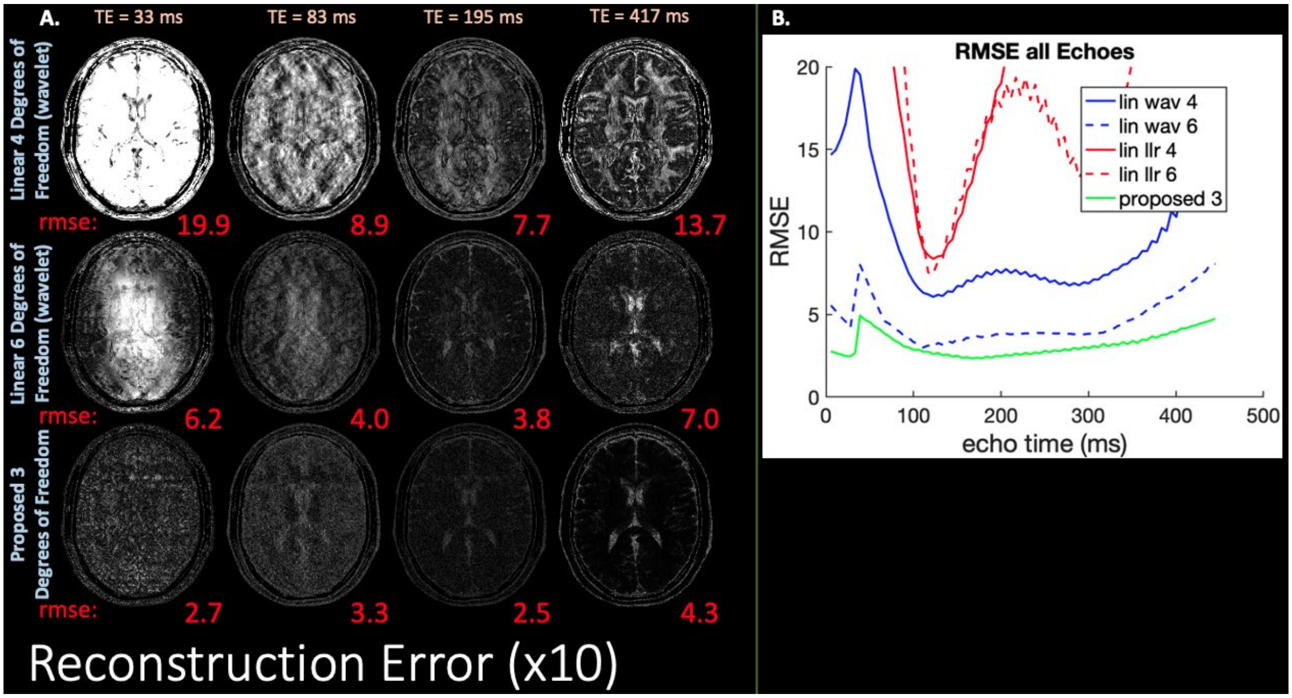

Supporting Information Figure S1

Retrospective in-vivo reconstruction experiments error maps and RMSE plots

Supporting Information Figure S2: Selected reconstruction (A) error maps and (B) RMSE across all echoes comparing the proposed approach and linear reconstructions on the retrospectively under-sampled, in-vivo T_2-shuffling dataset. The proposed framework reduces RMSE and image artifacts in comparison to the linear reconstructions.

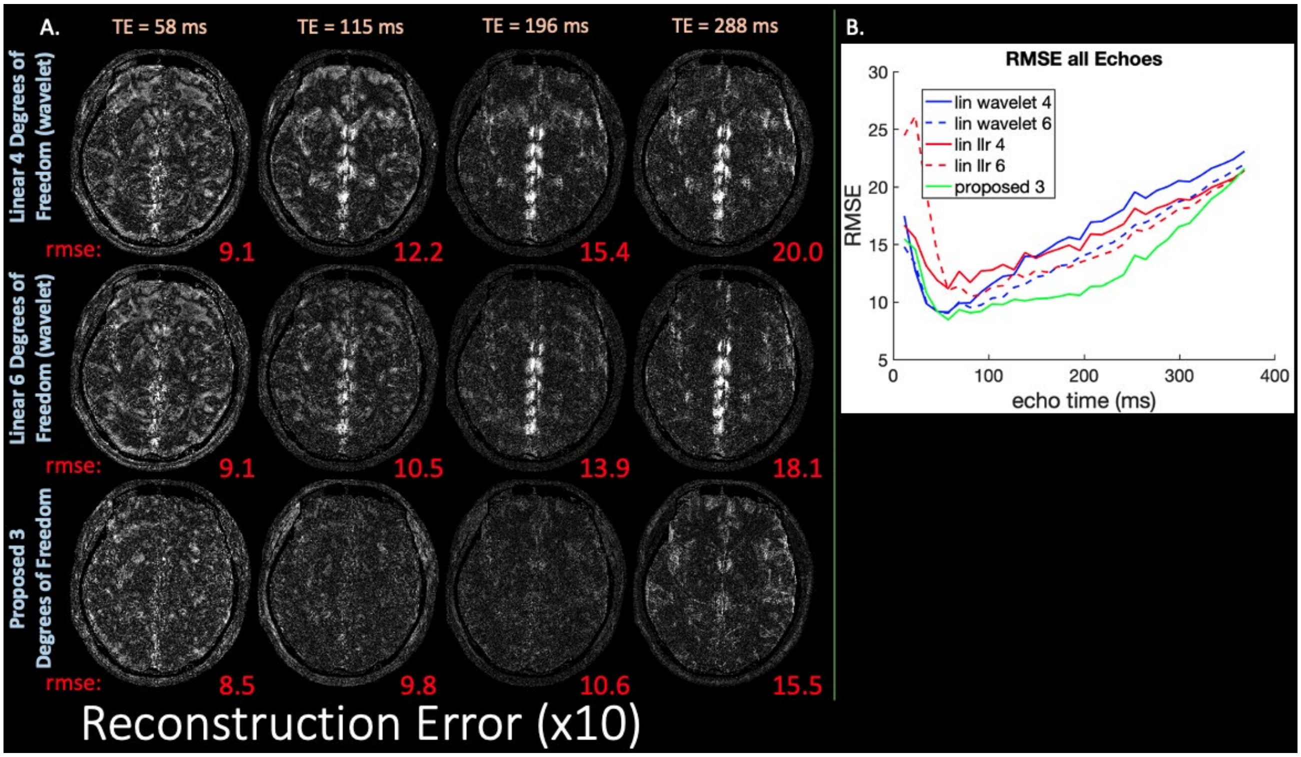

Supporting Information Figure S2

Retrospective Auto-encoder Hyperparameter Ablation Experiment

On a 8-shot dataset generated from the fully-sampled T_2-weighted dataset, an ablation study trained auto-encoders on combinations of the following hyper-parameters and compared the resultant un-regularized Latent Signal Model reconstructions.

- Nonlinearity: [tanh, leakyrelu]
- Training Epochs: [20K, 100K, 200K]
- Learning Rate: [1e-4, 1e-5]
- FC Layers: [2,3]
- Latent variables: [1]

Supporting Information Figure S3 compares the performance of different auto-encoder model hyper-parameters in in-vivo retrospective reconstructions. (A) and (B) display grids of average RMSE across all echoes for models with LeakyRelu and Tanh for a range of layers, learning rates, and epochs. LeakyRelu achieves lowest RMSE with 2 layers, 200K epochs, and 1e-5 learning rate, while Hyperbolic tangent achieves its minimum with 3 layers, 100K epochs, and 1e-4 learning rate. (C) Plots the performance of LeakyRelu and Tanh, with their respective best hyper-parameters, at 8 different random initializations. LeakyRelu varies significantly, while tanh yields consistent results.

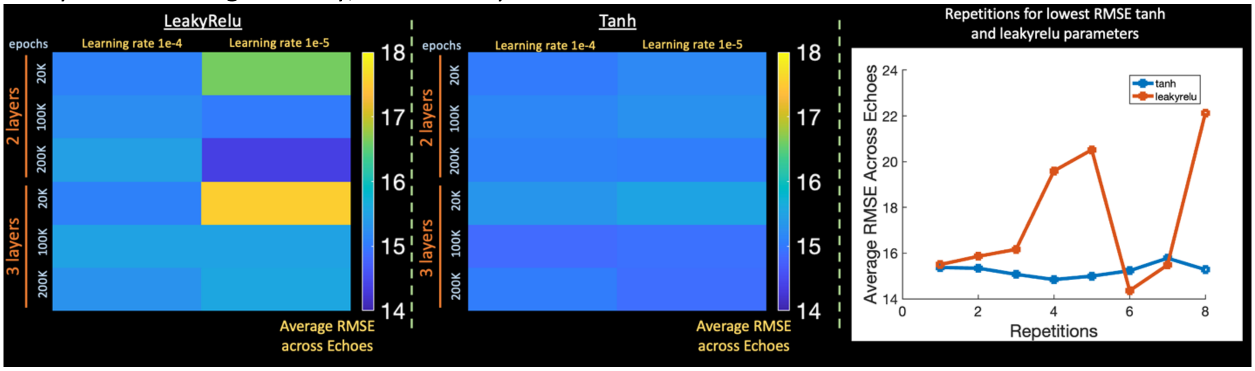

Supporting Information Figure S3

Simulation Experiments for analyzing the effects of reduced degrees of freedom and evaluating reconstruction stability across various noise instances

Supporting Information Figure S4: Linear and proposed reconstructions without regularization across 250 different k-space instances on the simulated T_2-shuffling dataset. The proposed approach achieves lower average absolute error maps and lower NRMSE across all the echoes, while maintaining comparable variance in reconstruction accuracy.

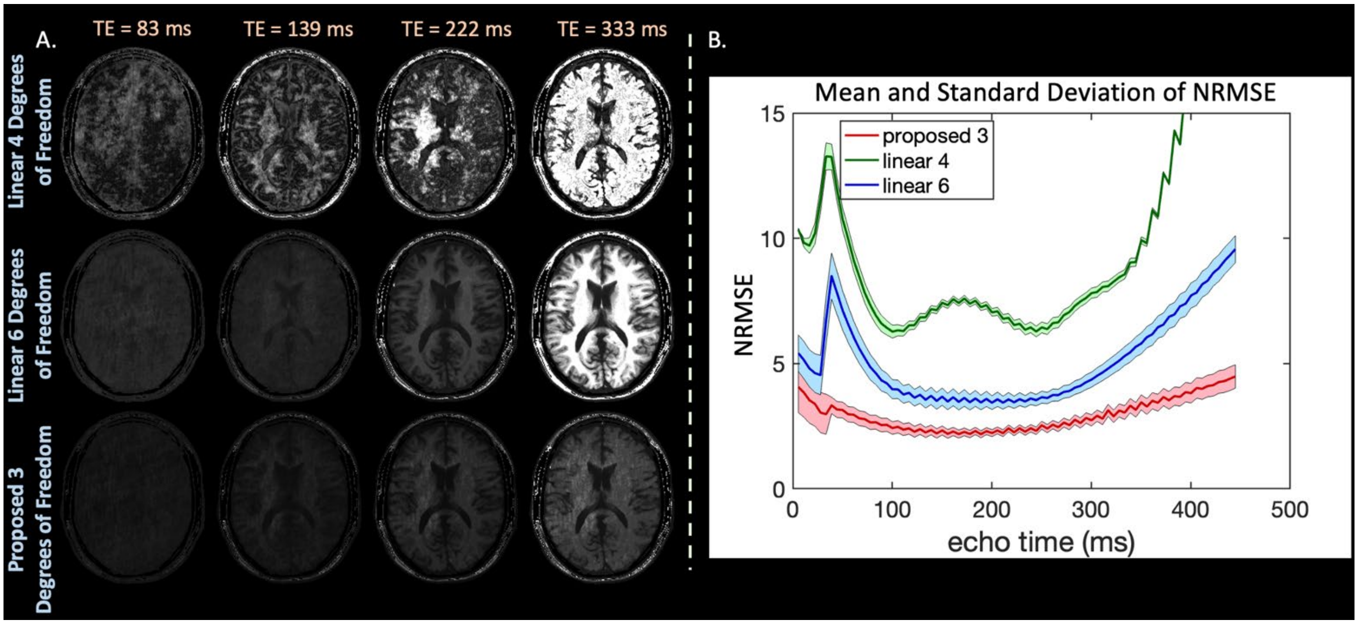

Supporting Information Figure S4

Latent Signal Models Finding a Solution of the EPG-based Forward Model on a Retrospectively Under-sampled In-vivo Dataset

Supporting Information Figure S5 (B) plots gradient norms with respect to T_2 and density of the EPG-based forward model as a function of the Latent Signal Model Optimization iteration on the retrospectively under-sampled in-vivo dataset. **Supporting Information Figure S5 (A)** displays exemplar reconstructed echo images from the proposed approach and the EPG-based forward model initialized with the proposed approach. The gradient norms approaching 2.7% of the maximum value and the quantitative similarity in the reconstructions suggest that the proposed approach efficiently finds a solution to the EPG-based forward model.

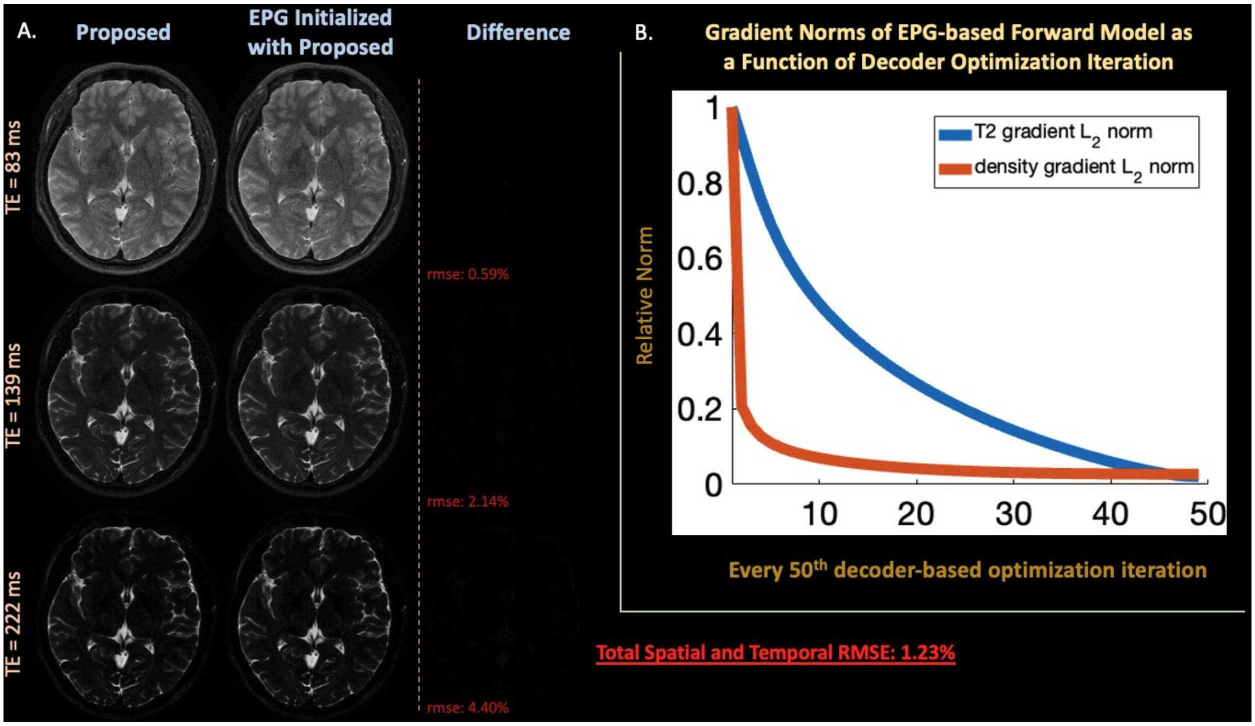

Supporting Information Figure S5

Gradient-Echo EPTI experiment errors and RMSE plots

Supporting Information Figure S6: Selected reconstruction (A) error maps and (B) RMSE across all echoes comparing the proposed approach and linear reconstructions on the retrospectively under-sampled EPTI dataset. The proposed framework reduces RMSE and image artifacts in comparison to the linear reconstructions.

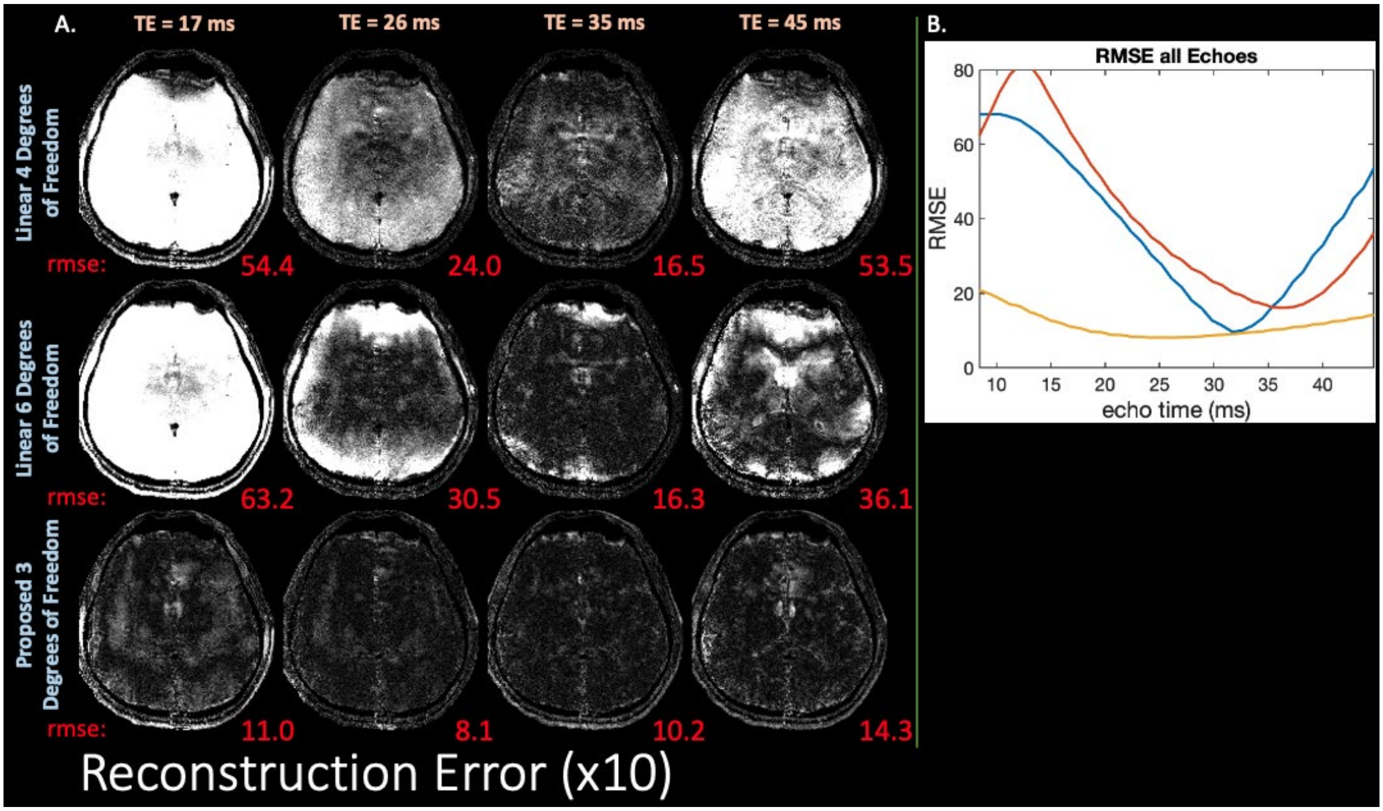

Supporting Information Figure S6

EPTI Reconstruction with Low-Resolution Phase

Supporting Information Figure S7: Similar comparisons of the proposed and linear subspace approach from Figure 7 using phase estimated from low-resolution calibration data in the GE-EPTI forward model. (A) displays exemplar echo images and associated error maps and (B) plots NRMSE for each echo. Even with a worse phase-estimate, the proposed approach still produces significantly higher quality images with lower NRMSE in comparison to the linear techniques.

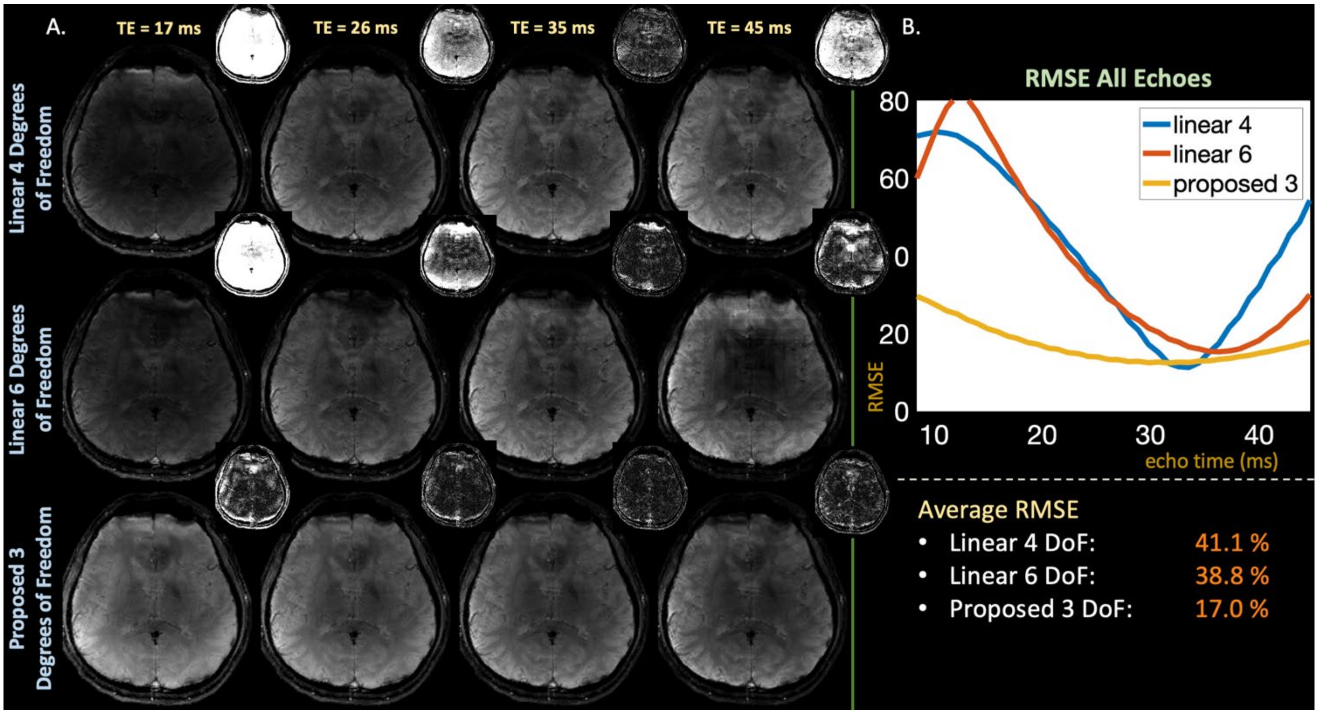

Supporting Information Figure S7

Partial Volume Experiments

Voxels at tissue boundaries produce some linear combination of signal from the multiple tissues present in the voxel. Linear subspace techniques implicitly model this effect as linear combinations of signals remain in the subspace. However non-linear models do not guarantee similar performance on linear combinations, so we perform three sets of experiments to evaluate Latent Signal Models in the presence of partial volume voxels.

- **a)** We generate a partial volume test set containing simulated linear combinations of single compartment signal from white matter, gray matter, and CSF to compare how well linear subspaces and Latent Signal Models represent both in- and out-of- phase mixtures.
- **b)** We examine whether Latent Signal Models exhibit higher reconstruction error at tissue boundaries in fully sampled in-vivo and simulated datasets.
- **c)** We compare selected, reconstructed inversion times and signal evolutions at CSF and Gray matter tissue boundaries in a less under-sampled, prospective, and in-vivo MPRAGE-shuffling experiment.

a) We begin by comparing how well linear subspaces and Latent Signal Models represent simulated linear combinations of signal evolution. Simulations generate single compartment signal S^A and S^B from two different tissues, and a linear combination produces partial volume signal, $S = \lambda^A S^A + \lambda^B S^B$. Let Φ represent a linear subspace and AE represent an auto-encoder both trained on a dictionary of simulated signal evolution without signal from voxels with partial volumes. We compare linear subspace reconstructions, $S_{linear} = \Phi\Phi^H S$, and Latent Signal Model autoencoder reconstructions, $S_{AE} = AE(S)$, to the ground truth signal S. Since these experiments only reconstruct simulated signal evolution and do not perform spatial reconstructions on imaging data, the auto-encoder does not require an additional complex scaling factor and the linear models do not require complex subspace coefficients. Thus, we compare auto-encoders with 1 latent variable to linear subspaces with 2, 3, and 4 real subspace coefficients and perform experiments with FSE and MPRAGE signal evolution.

We picked the following combinations of tissue parameters to represent partial volumes of white matter, gray matter, and CSF from FSE signal: ($T_2^A = 80\ ms, T_2^B = 110\ ms$), ($T_2^A = 80\ ms, T_2^B = 200\ ms$), and ($T_2^A = 110\ ms, T_2^B = 200\ ms$). We set $\lambda^A = \frac{1}{2}, \lambda^B = \frac{1}{2}$ to simulate in-phase additive mixtures and $\lambda^A = \frac{1}{2}, \lambda^B = -\frac{1}{2}$ to simulate out-of-phase subtractive mixtures.

Supporting Information Figure S8 plots estimated signal evolution from simulated, in-phase additive FSE partial volumes and resultant magnitude error with respect to the ground truth. While linear subspaces with 3 and 4 real coefficients achieve best results, the proposed auto-encoder achieves less than 4% RMSE for all partial volume linear combinations.

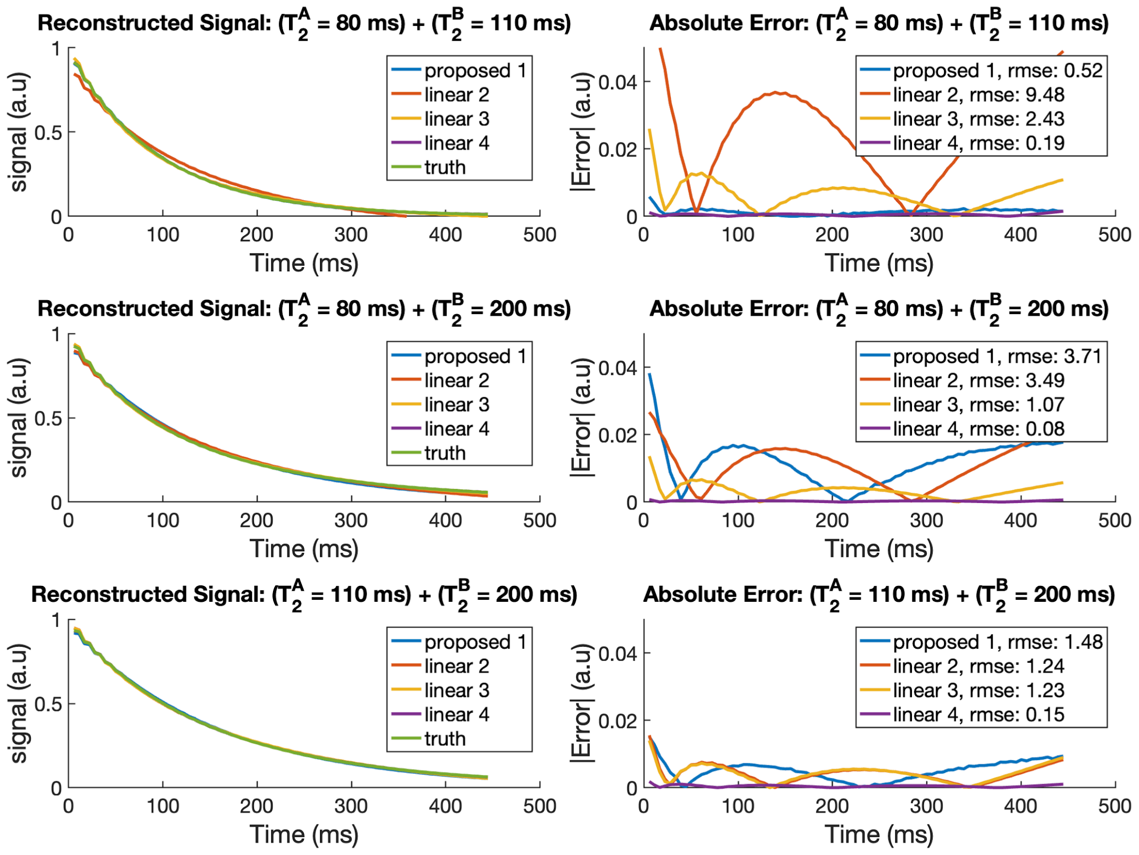

Supporting Information Figure S8

Supporting Information Figure S9 plots estimated signal evolution from simulated, out-of-phase subtractive FSE partial volumes and resultant magnitude error. Latent Signal Models represent signal poorly in comparison to linear subspaces with 3 and 4 real coefficients, which achieve less than 5% error. However, Latent Signal Models do not seem to exhibit worse performance at tissue boundaries in comparison to linear models in the in-vivo T_2 weighted experiments shown in Figure 4 of the main document. We suspect that this is due to the refocusing pulses in FSE sequences that induce minimal phase-variation within a single voxel resulting in additive partial volume mixtures, which Latent Signal Models do a much better job of representing.

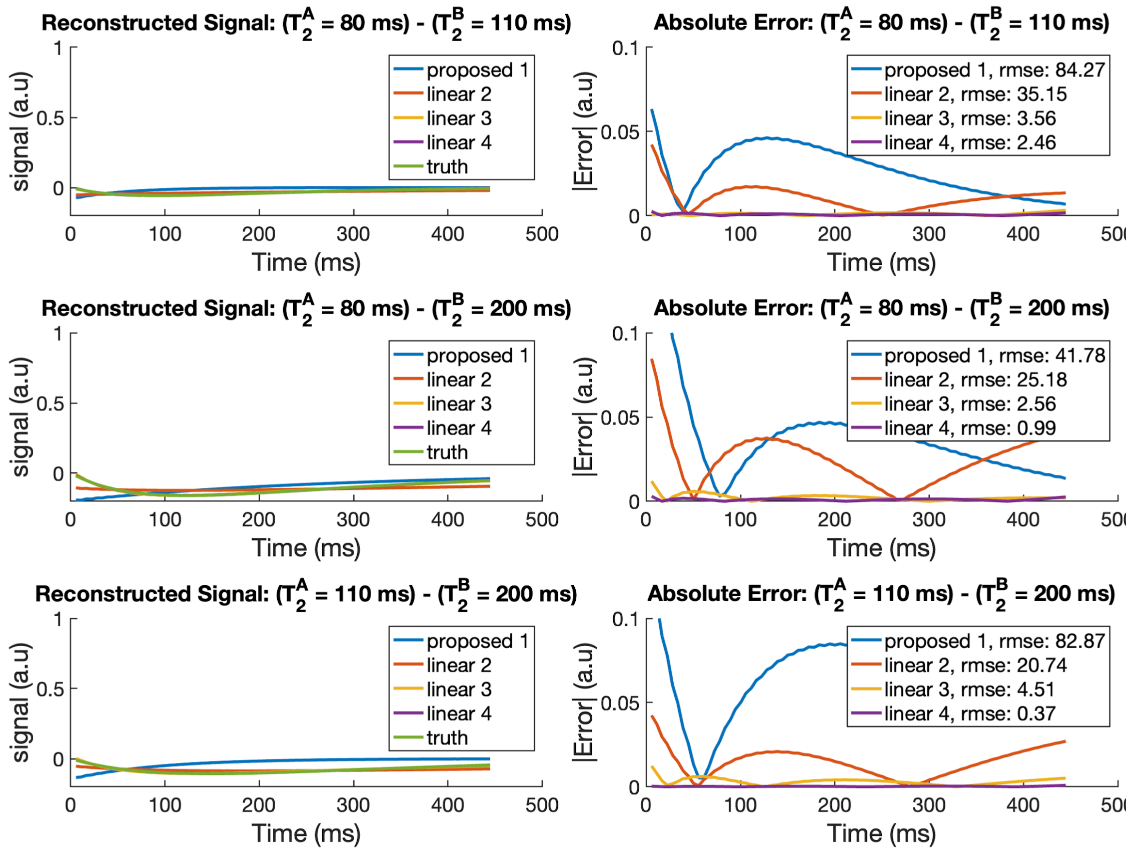

Supporting Information Figure S9

We similarly picked the following combination of tissues to represent partial volumes in MPRAGE signal and used the same values of λ^A and λ^B to represent in-phase and out-of-phase tissue: ($T_1^A = 831\ ms, T_1^B = 1351\ ms$), ($T_1^A = 831\ ms, T_1^B = 2201\ ms$), and ($T_1^A = 1351\ ms, T_1^B = 2201\ ms$).

Supporting Information Figure S10 plots estimated signal evolution from simulated, in-phase additive MPRAGE partial volumes and resultant magnitude error with respect to ground truth. All techniques represent signals within 0.5% RMSE.

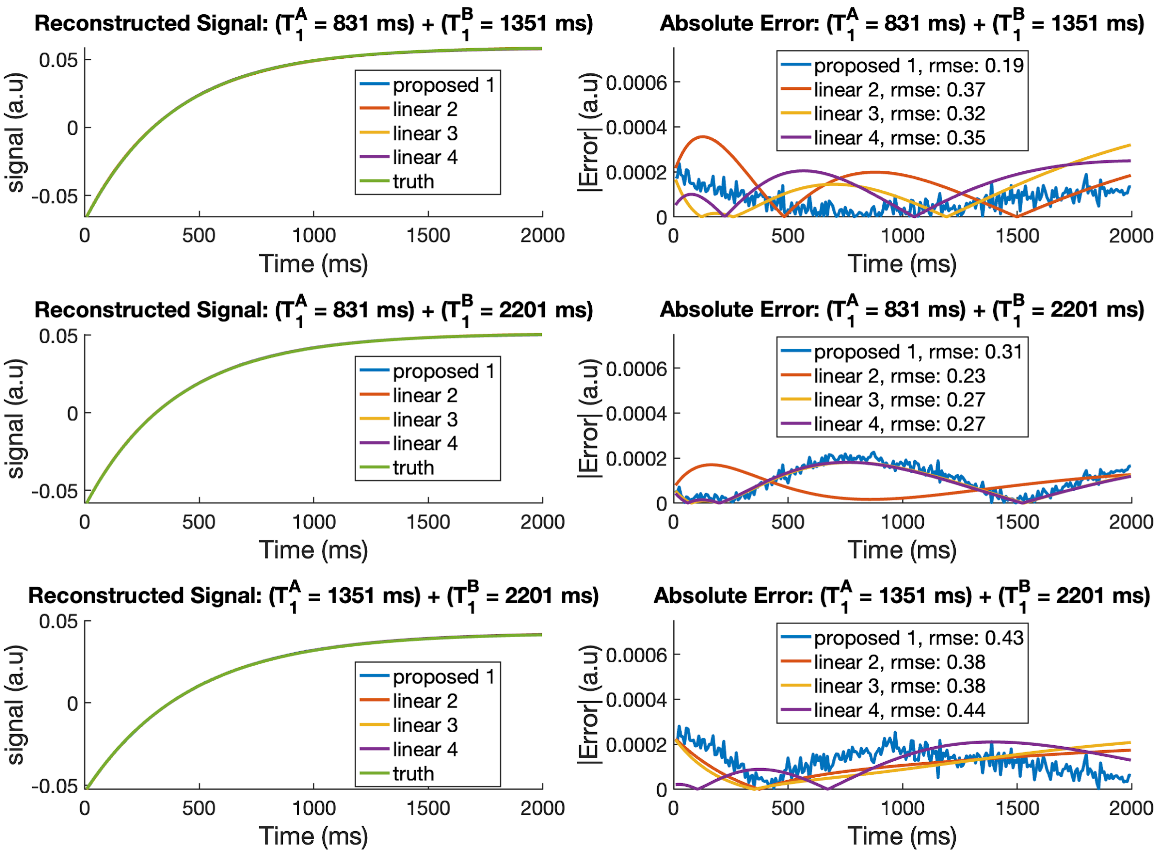

Supporting Information Figure S10

Supporting Information Figure S11 plots estimated signal evolution from simulated, out-of-phase subtractive MPRAGE partial volumes and resultant magnitude error. While the linear models achieve better performance, the proposed technique represents signal within 3.1% RMSE.

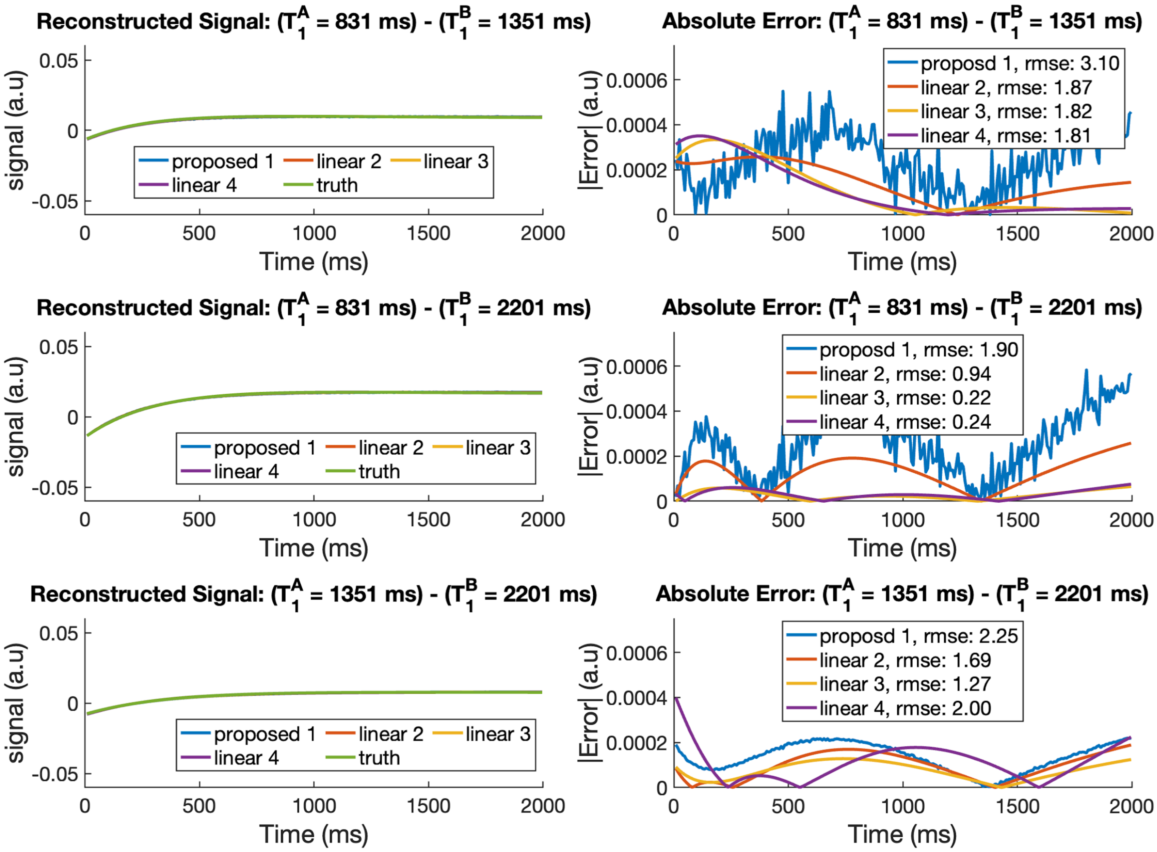

Supporting Information Figure S11

b) To further evaluate the effects of partial volumes in imaging datasets, we simulated acquisitions with partial volume effects. We generated imaging data for FSE and MPRAGE sequences and convolved images at every time-point with the kernel, $\frac{1}{5}(0\ 1\ 0\ 1\ 1\ 1\ 0\ 1\ 0\ )$, to simulate partial volumes with adjacent voxels. Then, fully sampled and under-sampled linear subspace and Latent Signal Model reconstructions produced images to evaluate sources of error. Since these experiments reconstruct imaging datasets, Latent Signal Models use 3 degrees of freedom (1 latent variable + real scaling + imaginary scaling) while linear models use 4, 6, or 8 degrees of freedom (2, 3, or 4 complex coefficients).

Supporting Information Figure S12 (A) shows reconstructions of selected echo times and corresponding error from fully sampled FSE reconstructions. Linear 4 suffer from bias due to the inability of 2 complex coefficients to adequately represent signal evolution and achieves 11.50% RMSE. Linear 6 and 8 achieve best performance with 1.44% and 0.55% RMSE respectively and exhibit noise-like error maps. The proposed approach achieves 2.03% average RMSE with higher error along tissue boundaries.

Supporting Information Figure S12 (B) displays selected echo times and corresponding error from under-sampled FSE reconstructions. Images from Linear 4 still exhibits bias with 10.10% average RMSE while Linear 8 suffers from noise amplification resulting in 16.20% average RMSE. Linear 6 balances noise amplification and signal representation with 5.83% average RMSE, but the proposed approach achieves best reconstruction with 4.14% average RMSE.

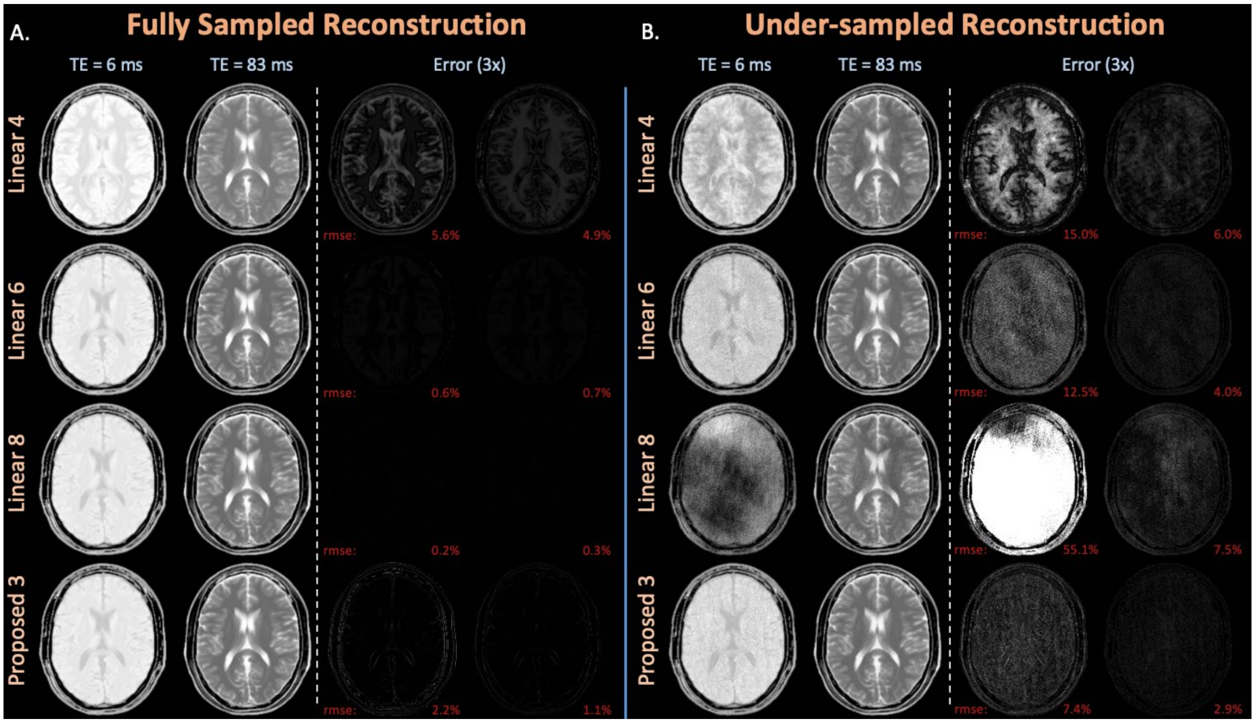

Supporting Information Figure S12

Supporting Information Figure S13 (A) shows reconstructions of selected inversion times and corresponding errors from fully sampled MPRAGE-shuffling data. The three linear models and the proposed approach all achieve high fidelity reconstructions with 0.77%, 0.56%, 0.64%, and 0.86% RMSE respectively. Linear models yield noise-like error maps, while the errors in Latent Signal Models concentrate near tissue boundaries.

Supporting Information Figure S13 (B) displays selected inversion times and error from under-sampled MPRAGE reconstructions. Noise amplification in the linear models yields 10.15%, 16.70%, and 23.51% average RMSE for 4, 6, and 8 degrees of freedom respectively. The proposed approach achieves less qualitative noise amplification and the lowest average RMSE of 8.86%.

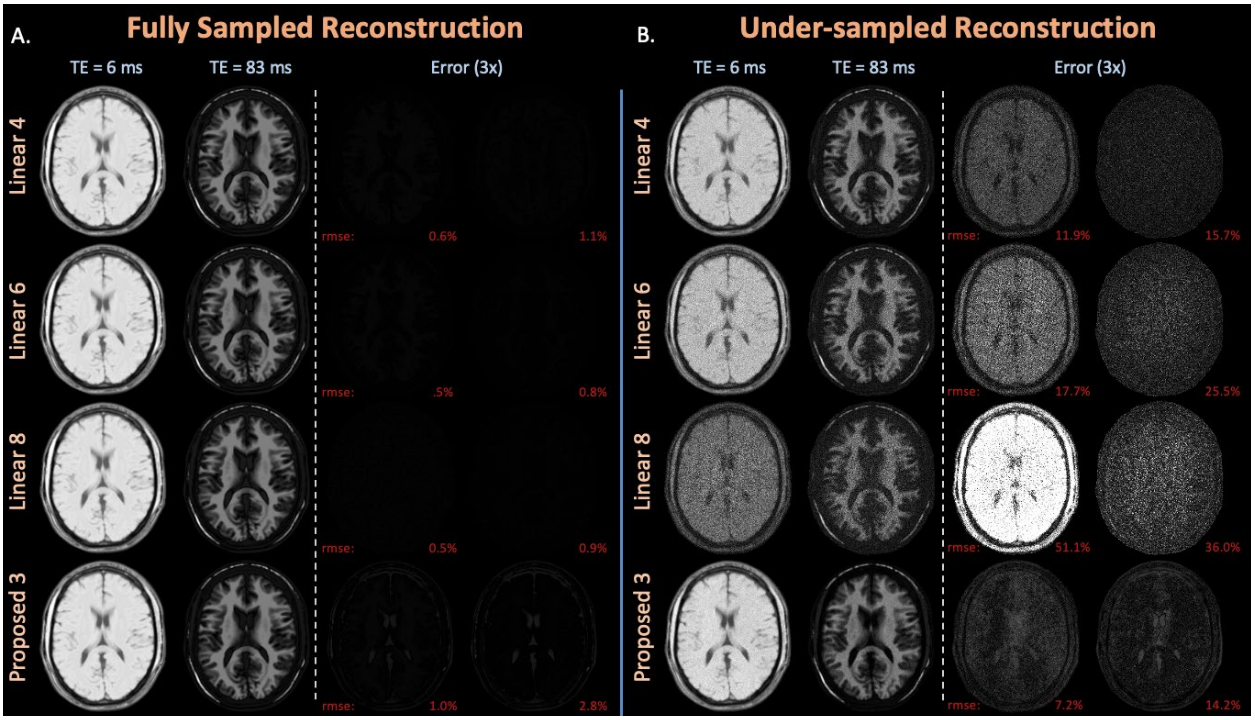

Supporting Information Figure S13

In **Supporting Information Figures S12 and S13,** linear models exhibit a tradeoff between accuracy of signal representation and reconstruction conditioning. In fully sampled experiments, linear models with 6 and 8 degrees of freedom out-perform Latent Signal Models through their ability to represent partial volumes more effectively. However, Latent Signal Models still achieve high fidelity reconstructions of 2% average RMSE in FSE data and 0.86% RMSE in MPRAGE data. In under-sampled experiments, noise amplification from reconstruction conditioning dominates as the source of error. Latent Signal Models achieve best performance as it represents partial volume signal well enough while also reducing noise-amplification more effectively in comparison to linear models.

c) The 3-spatial and temporal dimensions of the MPRAGE-shuffling sequence precludes acquisition of a fully sampled dataset. Towards a better understanding of partial volumes in in-vivo time-resolved MPRAGE reconstruction, we analyzed a less under-sampled, prospectively acquired dataset in comparison to what was used in the main manuscript. (Here we used an acquisition with R = 256, instead of R = 512)

Supporting Information Figure S14 shows selected inversion times from un-regularized linear subspace and Latent Signal Model reconstructions and compares temporal signal evolution magnitude and phase

from voxels on the gray matter / CSF boundary. Even with this less under-sampled acquisition, Latent Signal Models reduce noise amplification in comparison to linear reconstructions. Additionally, both the phase and magnitude of signal evolution in the partial volume voxels follow similar trajectories.

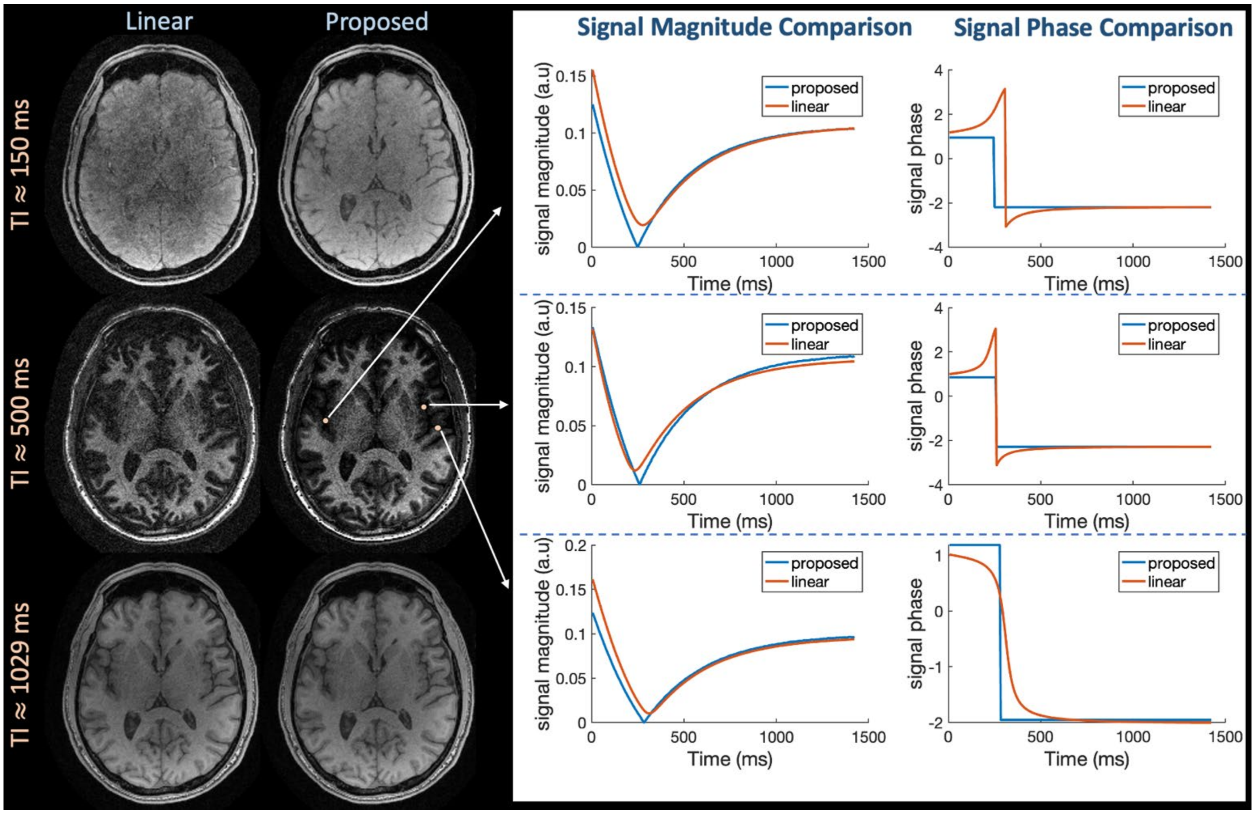

Supporting Information Figure S14

Relationship between Learned Latent Variables and T_1 or T_2

Supporting Information Figure S15 (A) plots the relationship between the learned latent variable, β versus T_2, for an auto-encoder trained on FSE signal evolution, while **Supporting Information Figure S3 (B)** plots β versus T_1 for MPRAGE signal evolution. Since our FSE and MPRAGE simulations vary as a function of one underlying parameter, the auto-encoder essentially learns a one-to-one 1D transformation between the latent variable and T_1 or T_2.

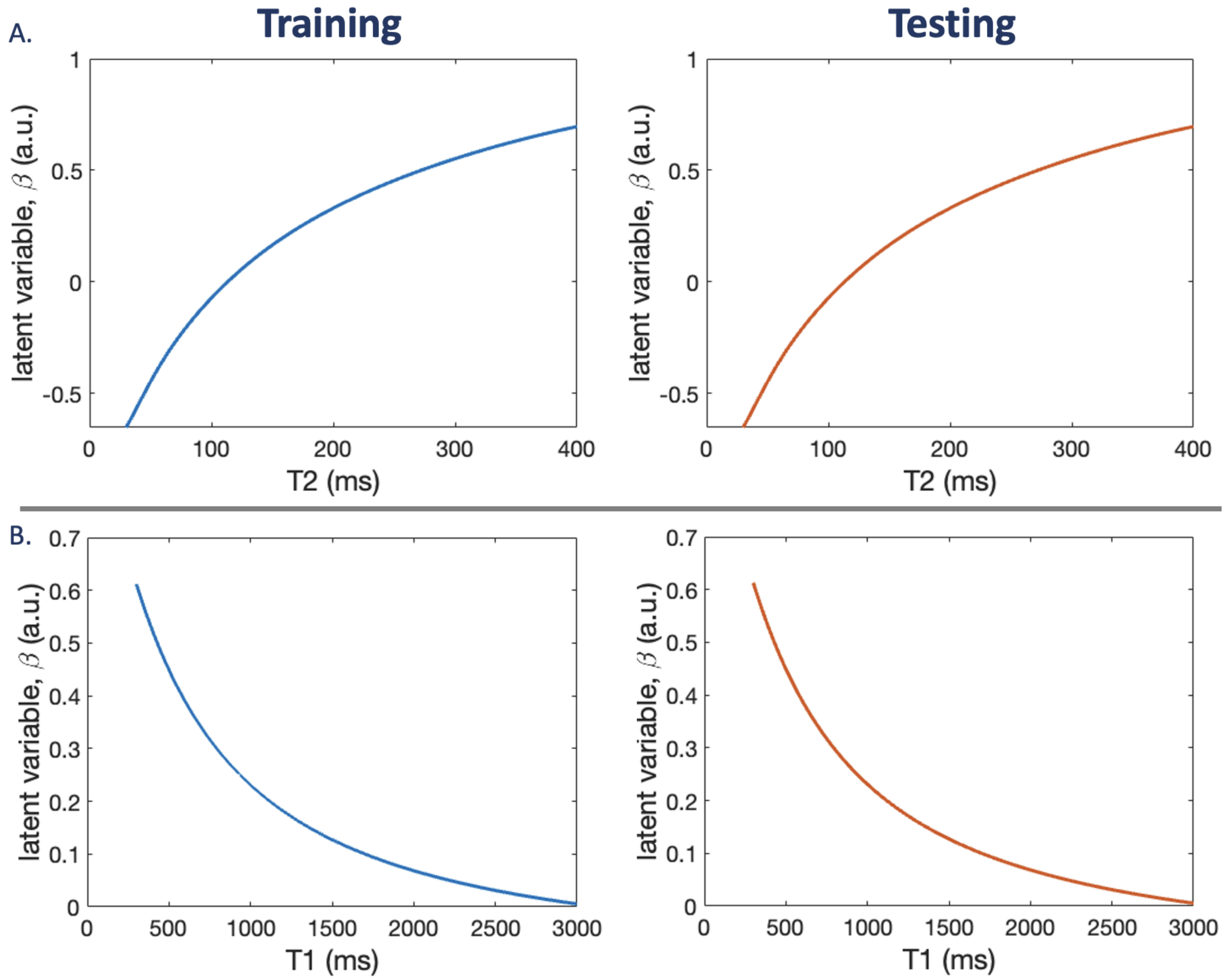

Supporting Information Figure S15

Deblurring Comparisons of Latent Signal Models and Linear Subspace Reconstructions

Linear subspace techniques, like T_2 shuffling in FSE applications, improve image sharpness by modeling signal dynamics that typically modulate k-space and produce blurring in non-time-resolved images. **Supporting Information Figure S16** evaluates whether Latent Signal Models also achieve sharpness improvements from a simulated 3D-FSE imaging dataset. The experiment compares a non-time-resolved reconstruction with center-out k-space sampling to a matching echo time image generated by linear and proposed time-resolved reconstructions using random k-space sampling. The time-resolved and non-time resolved methods acquired the same amount of data but employed different sampling schemes and reconstruction techniques. The non-time-resolved image suffers from blurring due to FSE signal decay modulating k-space. On the other hand, both the linear and proposed time-resolved techniques yield sharper images by modeling and reconstructing signal dynamics.

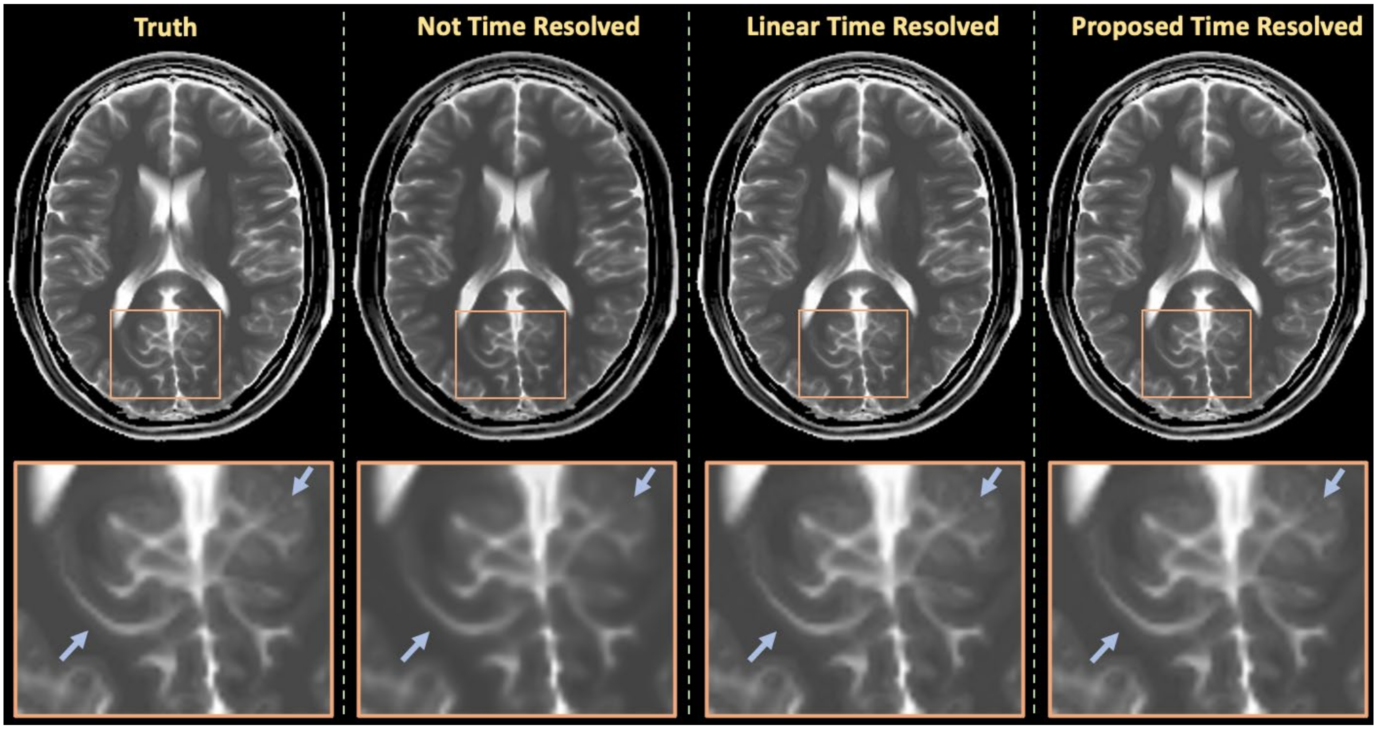

Supporting Information Figure S16
